# Correction to “Prospective Life Cycle Assessment of Lithium–Sulfur Batteries for Stationary Energy Storage”

**DOI:** 10.1021/acssuschemeng.4c01782

**Published:** 2024-04-05

**Authors:** Sanna Wickerts, Rickard Arvidsson, Anders Nordelöf, Magdalena Svanström, Patrik Johansson

In our original article, we assessed the environmental and resource impacts of a lithium–sulfur (Li–S) battery. We have found two errors in the end-of-life (EoL) modeling in that study:

- A calculation error concerning the ratio between primary and recycled lithium carbonate. This ratio was used to calculate how much primary lithium carbonate was needed in the Recycling and Combined scenarios.
- An erroneous calculation basis in the cell deactivation process, affecting all scenarios. The calculation basis did not account for the loss of cell mass in the deactivation properly.

The errors have been corrected, and new results have been generated for all scenarios and for the complete set of impact categories. Updated figures (Figure 4) and LCIA results in tabulated form (Table S41) are provided. Also, the affected unit processes have been corrected, see Tables S35, S36, and S38. In addition, we have updated Figure S17 to make it clearer how the closed-loop modeling of the recycled lithium carbonate was performed. Figures and tables belonging to the Supporting Information (SI) are provided in the updated SI file connected to this correction, while the figure belonging to the main article is presented below.

**Figure 4:**
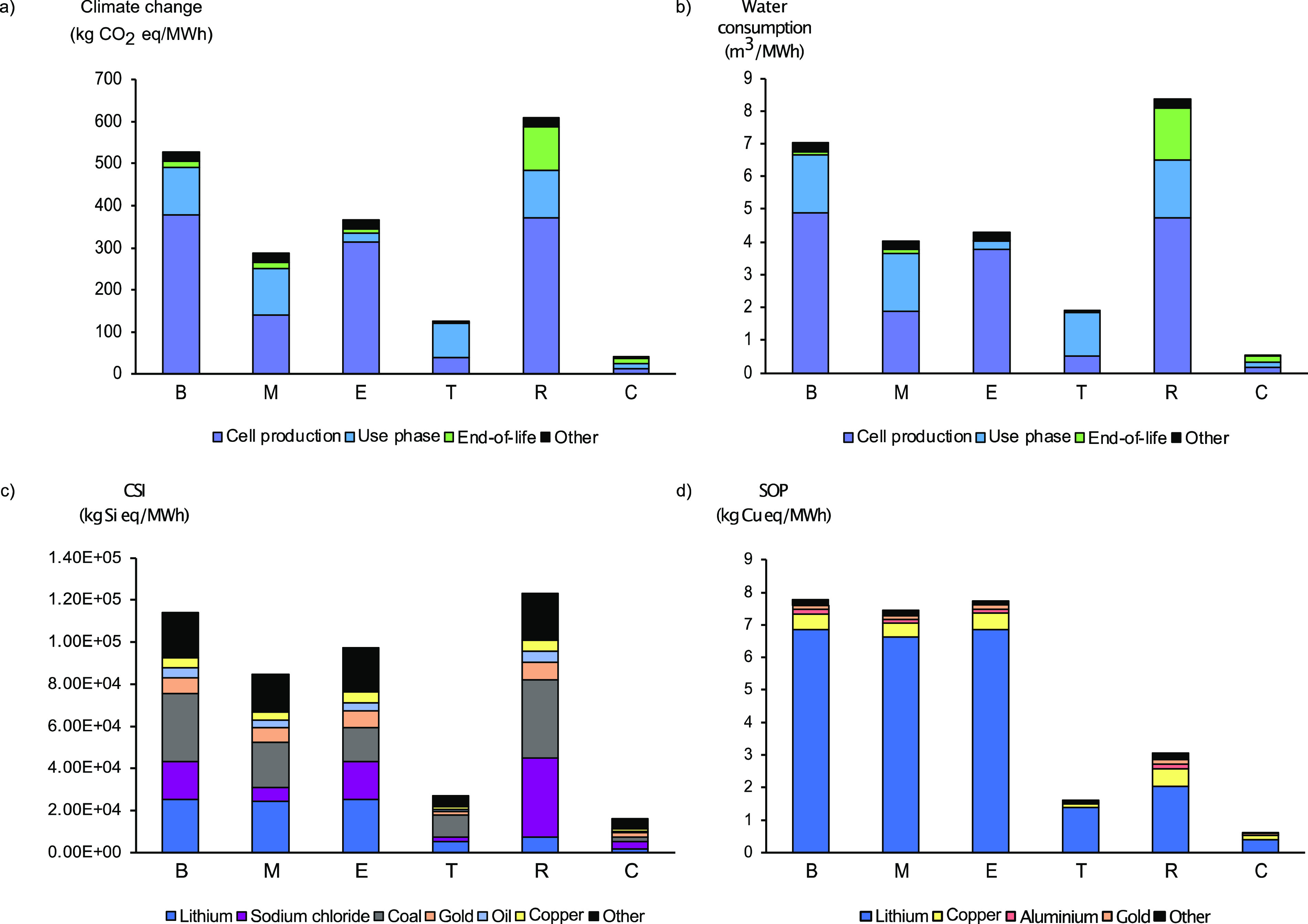
Cradle-to-grave results for (a) climate change, (b) water consumption, (c) the crustal scarcity indicator (CSI), and (d) the surplus ore potential (SOP) of the Li–S battery used for stationary energy storage. The functional unit is 1 MWh of electricity delivered to the grid over 20 years. B = base scenario, M = material selection scenario, E = energy system scenario, T = technical performance scenario, R = recycling scenario, and C = combined scenario.

The corrected results are similar to the previously published results. The only notable changes are lower surplus ore potential (SOP) indicator results from hydrometallurgical recycling of lithium carbonate in the corrected results. This is because our original article erroneously considered 58% primary and 42% recycled lithium carbonate. The correct shares, however, are 30% and 70%, respectively. Since lithium extraction has a large influence on the SOP indicator results, the lower share of primary lithium carbonate (from 58% to 30%) reduces the SOP indicator results notably.

## Supporting Information Available

Corrected Tables S35, S36, S38, and S41 and corrected Figure S17 (PDF)

