# Supplementary material for: Correction to “Prospective Life Cycle Assessment of Lithium–Sulfur Batteries for Stationary Energy Storage”
Source: ACS Sustain Chem Eng. 2024 Apr 5;12(15):6035–6. doi: 10.1021/acssuschemeng.4c01782 (PMC11022237; doi:10.1021/acssuschemeng.4c01782)
Supplement: Supplementary file 1 — sc4c01782_si_001.pdf [file sc4c01782_si_001.pdf]

SUPPORTING INFORMATION

Prospective life cycle assessment of lithium-sulfur batteries for stationary energy storage

Sanna Wickerts*[1], Rickard Arvidsson[1], Anders Nordelöf[1,2], Magdalena Svanström[1], Patrik Johansson[3,4]

[1] Environmental Systems Analysis, Chalmers University of Technology, Vera Sandbergs Allé 8, 412 96 Gothenburg, Sweden.

[2] Institute of Transport Economics, Gaustadalléen 21, 0349 Oslo, Norway.

[3] Materials Physics, Department of Physics, Chalmers University of Technology, 41296 Gothenburg, Sweden.

[4] ALISTORE – European Research Institute, Rue Baudelocque, Amiens 80000, France

*Corresponding author. Phone: +46 (0)706847799. E mail: sanna.wickerts@chalmers.se

Number of pages: 62

Supplementary figures: 18

Supplementary tables: 41

Table of Contents

S1 Comparison between LCAs of Li-S batteries ... *S1*

S2 Prospective modeling of cell materials and recycling ... *S3*

S3 Unit process modeling ... *S5*

S3.1 LiTFSI production ... S5
S3.1.1 CF_3SO_2F production ... S7
S3.1.2 CF_3SO_2NHNa production ... S8
S3.1.3 $(Me_3Si)_2NH$ production ... S9
S3.1.4 $CF_3SO_2NNaSiMe_3$ production ... S11
S3.1.5 $(CF_3SO_2)_2NNa$ production ... S12
S3.1.6 $(CF_3SO_2)_2NH$ production ... S13
S3.1.7 $(CF_3SO_2)_2NLi$ production ... S14

S3.2 Lithium triflate production ... S16
S3.2.1 CF_3SO_3Na and CF_3SO_3H production ... S17
S3.2.2 $LiCF_3SO_3$ production ... S19

S3.3 Polyehtylene glycol production ... S20

S3.4 Sulfolane production ... S22

S3.5 Mesoporous carbon production ... S24
S3.5.1 Pluronic P123 production ... S25
S3.5.2 SBA-15 production ... S26
S3.5.3 CMK-3 production ... S27

S3.6 Cell production ... S28
S3.6.1 Lithium foil production ... S29
3.6.2 Separator production ... S30
S3.6.3 Cathode production ... S32
S3.6.4 Electrolyte mixing and feeding ... S35
S3.6.5 Cell assembly and formation ... S36
S3.6.6 1 kWh storage capacity ... S38

S3.7 Battery module production ... S39

S3.8 BMS production ... S41

S3.9 Battery rack production ... S43

S3.10 Fire suppression system production ... S44

S3.11 Installation production ... S45

S3.12 Use phase ... S47

S3.13 Disassembly and end-of-life treatment ... S49
S3.13.1 Shredding and sorting ... S51
S3.13.2 Cell deactivation ... S52
S3.13.3 Cell separation and grinding ... S53
S3.13.4 Landfilling ... S54
S3.13.5 Hydrometallurgical treatment ... S55

S3.14 Electricity supply ... S60

S4 LCIA results ... *S60*

References ... *S62*

S1 Comparison between LCAs of Li-S batteries

To the best of our knowledge, seven previous life cycle assessment (LCA) studies of lithium-sulfur (Li-S) batteries have been conducted: Deng et al. [1], Arvidsson et al. [2], Cerdas et al. [3], Wolff et al. [4], Lopez et al. [5] Benveniste et al. [6] and Barke et al. [7]. These seven studies are compared to the present study in Table S1. Manufacturing readiness levels (MRLs), provided in the table are based on the scale in and estimated by the authors of this study.[8]

Table S1. Modeling choices of LCAs assessing Li-S batteries. CB = carbon black, CNT = carbon nanotubes, DOL = 1,3-dioxolane, DME = dimethoxyethane, EC = ethylenecarbonate, MRL = manufacturing readiness level, LiTFSI = lithium bis(trifluoromethanesulfonyl)imide, LiOTf = lithium triflate, PE = polyethylene, PEG = polyethylene oxide, PP = polypropylene, PVDF = polyvinylidene fluoride, PVP = polyvinylpyrrolidone, SL = sulfolane.

Modeling aspect	This study	Deng et al.,[1]	Arvidsson et al.,[2]	Cerdas et al.,[3]	Wolff et al.,[4]	Lopez et al.,[5]	Benveniste et al.,[6]****	Barke et al., [7]
**Anode**	Lithium foil	Lithium foil	Lithium foil	Lithium foil	Lithium foil	Lithium foil	Lithium foil	Lithium foil
**Cathode**	Sulfur + CMK-3 + PVDF / Sulfur + CB + PEG	Graphene oxide + Sodium thiosulfate+PVP + CB + PVDF	Sulfur + CB/CNT/ graphene*	Sulfur + CB + PVDF	Sulfur + carbon + PVDF	5 novel cathode materials***	Sulfur + carbon + PVDF	Sulfur + CB + solid state based + solid electrolyte
**Electrolyte**	LiTFSI + $LiNO_3$ + DOL + DME / LiOTf + SL	LiTFSI + $LiNO_3$ + DOL + DME	LiTFSI + $LiNO_3$ + DOL + DME	$LiPF_6$ + EC	LiTFSI + DOL + DME	LiTFSI + $LiNO_3$ + DOL + DME	LiTFSI + DOL + DME	$Li_{10}Ge(PS_6)_2$/$Li_{10}Sn(PS_6)_2$/ $Li_{10}Si(PS_6)_2$/Li_6PS_5Cl
**Separator**	PE + PP	PP + PE	PP + PE	PP	PP + PE	PP	PP + PE	$Li_{10}Ge(PS_6)_2$ + polyvinylfluoride
**Current collector**	Al	Al, Cu	Al	Al, Cu	Unclear	Al	Unclear	Al, Cu
**Cell type**	Pouch	Pouch	Unclear	Pouch	Coin	Unclear	Coin	Pouch
**Specific energy density (Wh/kg)**	150 or 500 (cell level)	220 (pack level)	300 or 500 (cell level)	303 ("stack level")	295 (cell level)	Unclear	295 (cell level)	644-813
**System boundary**	Cradle-to-gate / cradle-to-grave	Cradle-to-grave	Cradle-to-gate	Cradle-to-grave	Cradle-to-grave	Cradle-to-gate	Cradle-to-grave	Cradle-to-gate
**Battery application**	Large scale stationary energy storage	Electric vehicle	Not specified	Electric vehicle	Electric vehicle	Not specified	Electric vehicle	Intended for electric aircrafts
**Allocation approach**	Mass based	Unclear	Economic/mass/none	Unclear	Unclear	Unclear	Unclear	Unclear
**Electricity supply**	EU mix / wind	US country mix	Solar / coal**	CN / JP / KR country mix	EU mix	EU mix	EU mix	CN / DE
**Modeled MRL**	Industrial scale, MRL = 10	Pilot / industrial scale, MRL ~ 6-9	Pilot / industrial scale, MRL ~ 6-9	Pilot scale, MRL ~ 6-8	Pilot / industrial scale, MRL ~ 6-9	Laboratory scale, MRL ~ 3-5	Pilot / industrial scale, MRL ~ 6-9	Industrial scale, MRL = 10

*No binder included

**Changed in the background system as well

***Colloidal sulfur + CB + NaCMC, sulfur + CNT + Co + graphene + PVDF, Li_2S + graphene + PVDF, sulfur + graphene + MgB_2 + PVDF, and sulfur + CoS_8 + PVDF-Super P

**** This study report using the same modelling and technology choices as Wolff et al. (2019).

S2 Prospective modeling of cell materials and recycling

In order to model a future industrial-scale Li-S battery production scenario, the framework by Piccinno et al. [9] is used for scaling up the production of upstream emerging battery materials and the Li-S battery recycling process. Input and output amounts for these processes are obtained from laboratory-scale synthesis descriptions as found in patents, experimental studies and other LCA studies. If some input and/or output values are not provided in the synthesis description, stoichiometric calculations are used to obtain those inputs and/or outputs. A 20% reduction of solvent amount is likely for a scaled-up process,[9] which is thus applied for all modeled processes where solvents are applied. Individual process steps, such as heating and stirring, are identified in the synthesis descriptions, after which the corresponding equations in the upscaling framework are used to calculate their energy requirements. The equations mostly used in this study are those for heating (Eq. S1) and stirring (Eq. S2). In Eq. S1, the specific heat capacity (C_p) of the reaction mixture, the total mass of the reaction mixture (m_{mix}), the reaction temperature (T_r) and the reaction time (t) are needed. In terms of the specific heat capacity, this is approximated using the solvent's C_p or the C_p for the reagent in excess. In Eq. S2, the density of the reaction mixture (ρ_{mix}) and the reaction time are needed. The solvent or the reagent in excess is used to approximate the density of a mixture. Drying is also mentioned in some synthesis descriptions, for which Eq. S3 is used to calculate the energy requirement. In this case, the C_p for the liquid to be dried, the input liquid mass to the drying process (m_{liq}), the boiling point of the liquid (T_{boil}) as well as the starting temperature (T_0), the enthalpy of vaporization (ΔH_{vap}) and the evaporated mass (m_{vap}) need to be provided. The efficiency of the dryer (η_{dry}) is set to 80% by default according to Piccinno et al. [9]. Values for C_p and ΔH_{vap} are again approximated based on the solvent or the chemical in excess. The m_{liq} parameter needs to be approximated if no such input is stated in the synthesis description. Provided that there have been prior liquid removal processes, such as distillation, it is assumed that 10% of the original liquid mass needs to be dried off. Furthermore, a 100% liquid removal is assumed unless the evaporated mass is stated in the laboratory description.

$$Q_{react(1000l)} = \frac{Q_{heat} + Q_{loss}}{\eta_{heat}} = \frac{C_p \cdot m_{mix} \cdot (T_r - 298.15\ K) + 3.303 \cdot (T_r - 298.15\ K) \cdot t}{0.75} \quad \text{(Eq. S1)}$$

$$E_{stir\ (1000l)} = 0.018 \cdot \rho_{mix} \cdot t \quad \text{(Eq. S2)}$$

$$Q_{dry} = \frac{C_{p,liq} \cdot m_{liq} \cdot (T_{boil} - T_0) + \Delta H_{vap} \cdot m_{vap}}{\eta_{dry}} \quad \text{(Eq. S3)}$$

In some synthesis descriptions, grinding and filtration are reported, for which ranges of energy requirements of the corresponding upscaled process are provided in Piccinno et al. [9]. The average values of these ranges, i.e. 0.012 kWh/kg grinded material and 0.0055 kWh/kg dry material (i.e., per kg filter cake), are used for estimating the grinding and filtration energy requirement. In addition, rotary evaporation is mentioned in some synthesis descriptions. In this case, distillation is assumed as a proxy process, for which 1.5 kg steam/kg waste solvent and 27 kg cooling water/kg waste solvent are used as proxy values. These values are provided by Piccinno et al. [9] but originally obtained by Capello et al. [10].[1] Here, we apply these values per kg of material to be distilled, since that corresponds to the amount of waste solvent in the proxy data. Distillation is also mentioned as an individual process in several synthesis descriptions, for which the same proxy values for steam and cooling water are used per kg of waste solvent. If no information about the energy source is provided in the laboratory-scale description, fossil-based steam is assumed as the energy source for heating and drying, whereas electricity (sourced from different supplies as described in Section S3.14) is assumed as energy source for mixing, grinding and filtration.

If there are additional products besides the main product, we follow the definition of co-products and by-products in Piccinno et al. [9]: Co-products are reaction products having economic value similar to the main product, while by-products are reaction products that have considerably lower economic value. In the case of co-products, partitioning of the environmental burden between the main product and co-product(s) is conducted using mass-based allocation.[2] In the case of by-products, due to their low current and anticipated future value, these are assumed to become waste, following liquid waste streams of e.g. spent solvents. Regarding recovery, organic solvents are assumed to be recycled using distillation. When water is used as a solvent, it is assumed not to be recycled. The amount of waste generated is calculated from mass balances. Water-based waste streams are assumed to be sent to wastewater treatment and liquid organic waste streams are modeled as "treatment of spent

[1] Here, a detail can be noted: Piccinno et al. (2016) provide a value of 0.027 kg/kg waste solvent, citing Capello, *et al* [9], but the original value from Capello, *et al* [9] is 0.027 m^3/kg waste solvent. The original value is therefore used in this study.

[2] It can be noted that the product system studied only includes one co-product: Trimethylsilyl chloride (Me_3SiCl), which is used in as an input to the hexamethyldisilazane production (see Section S3.1.3).

solvent". Cooling water, used in processes such as distillation and refluxing, is also assumed to be sent to a wastewater treatment plant after use.

S3 Unit process modeling

In the following subsections (Section S3.1-S3.14), the modeling of all unit processes that have been developed in this study is described, as well as for unit processes obtained from the literature but significantly modified. We follow the basic steps for unit process development described by Zhang et al. [11], along with the optional step to match flows with background datasets. For each unit process, there is thus a description, a flowchart and a table where the inputs and outputs of the unit process are reported. Most unit processes are scaled up from laboratory production descriptions by applying the upscaling framework from Piccinno et al. [9], described in Section S2. There are some unit processes where the input values differ depending on the scenario, which is why several input and output values are reported in some tables. If only one input value of a parameter is reported in a certain table, that parameter value applies to all scenarios.

S3.1 LiTFSI production

Lithium bis(trifluoromethanesulfonyl)imide (LiTFSI) has a complex synthesis route with several reaction steps, see Figure S1. Desmarteau and Witz [12] describe this route with trifluoromethanesulfonyl fluoride (CF_3SO_2F) as starting material. For some of the compounds along the route, no common chemicals names are available. In those cases, we follow Desmarteau and Witz [12] and use the molecular formulas in the process descriptions. In addition, the chemical reactions provided by Desmarteau and Witz [12] are not balanced. Since no co-products are assumed to be produced in the LiTFSI synthesis, the chemical reactions in Desmarteau and Witz [12] are therefore described in a simplified way here, with by-products aggregated into one joint reaction product:

reactant A + reactant B $\longrightarrow$ main product C + by-products

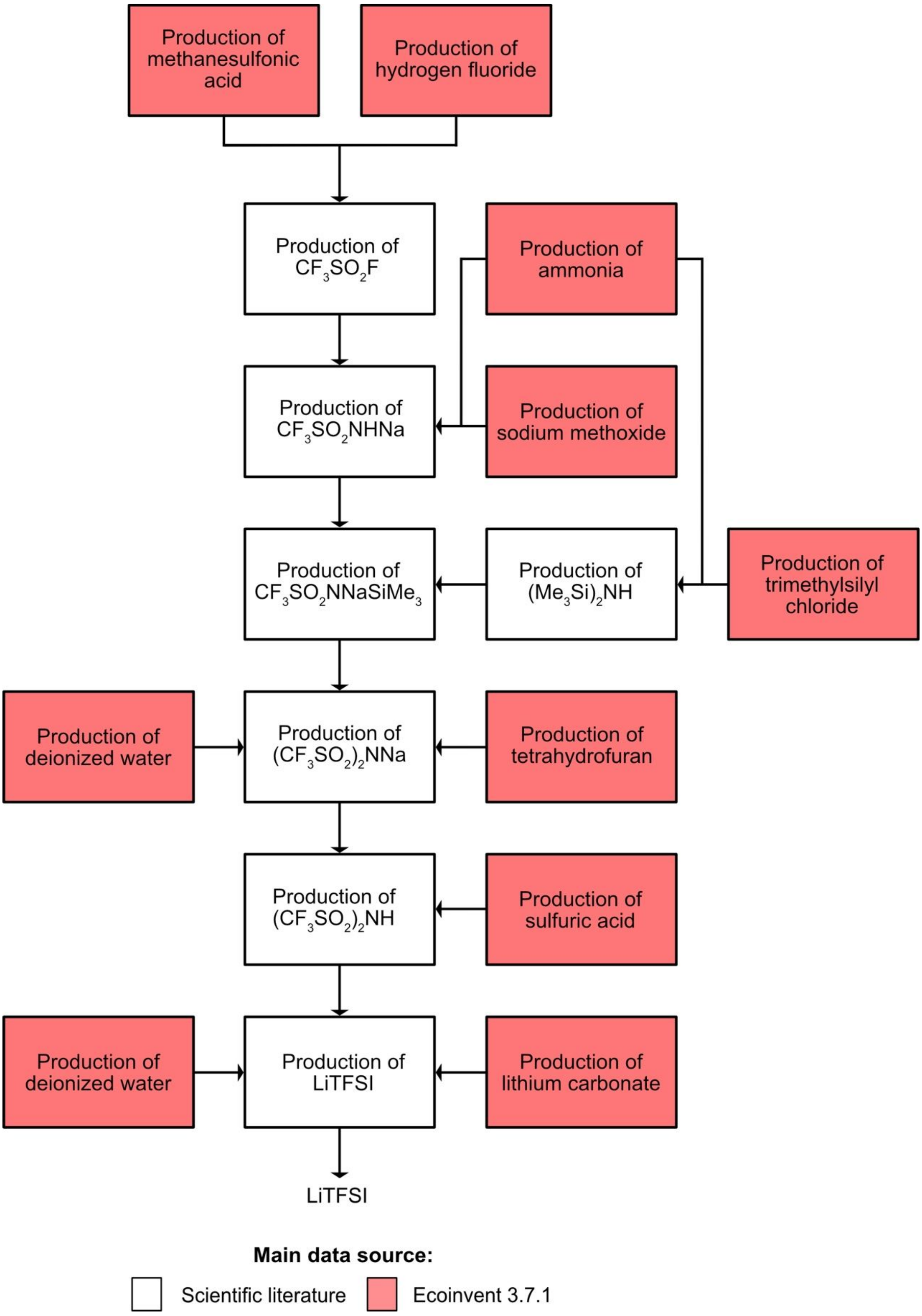

Figure S1. Flowchart of LiTFSI production.

S3.1.1 CF_3SO_2F production

The modeled production system for LiTFSI starts with the production of trifluoromethanesulfonyl fluoride (CF_3SO_2F), which is industrially produced by electrochemical fluorination of methanesulfonic acid with anhydrous hydrogen fluoride,[13] see SR1. Electrochemical fluorination is carried out by electrolysis. Stoichiometry is used to calculate the input amounts, using a generic reaction yield of 95% since no specific yield is reported for that reaction.[14] Energy requirements for the fluorination process could not be found, thus the electricity requirement for electrolysis of lithium chloride serves as a proxy.[15] Mass balance is used to calculate the amount of waste consisting of unreacted reactants. As the reaction mixture contains organic components, the generated waste is assumed to undergo treatment of spent solvents by incineration. The unit-process table for this process is presented in Table S2.

$$CH_3SO_3H + 4HF \longrightarrow CF_3SO_2F + H_2O + 3H_2 \qquad \text{(SR1)}$$

Table S2. Unit-process table for CF_3SO_2F production.

Material inputs	Normalized value	Unit	Upstream data source
Methanesulfonic acid	6.6E-1	kg	market for methane sulfonic acid - GLO
Hydrogen fluoride	5.5E-1	kg	market for hydrogen fluoride - RoW
Energy and process inputs			
Electricity	3.0E1	kWh	Scenario dependent, see Table S39
Facility inputs			
Chemical factory	4.0E-10	item	market for chemical factory, organics - GLO
Product output			
Trifluoromethanesulfonyl fluoride	1.0E0	kg	-
Waste outputs			
Organic waste	2.1E-1	kg	market for spent solvent mixture - RoW

S3.1.2 CF_3SO_2NHNa production

The compound CF_3SO_2NHNa can be synthesized by letting trifluoromethanesulfonyl fluoride first react with liquid ammonia and then reacting the intermediate product with sodium methoxide in methanol.[12] The net reaction can be seen in SR2. The authors report all input amounts and a reaction yield of 95%. Regarding the first part of the reaction, a temperature of -78ºC is necessary since the ammonia must be in a semi-frozen state. Piccinno et al. [9] do not provide equations for calculating energy requirements for cooling. The energy demand for this process step is therefore estimated by assuming the same energy requirement as for heating the mixture to +78ºC using Eq. S1. For the next part of the reaction, processes such as heating, filtration and rotary evaporation are reported. Eq. S1 for heating and the average value for filtration are used to obtain energy requirements.[9] Rotary evaporation is approximated as distillation, and since the data required for obtaining the energy demand and cooling water requirements are not found, the proxy values 1.5 kg of steam/kg solvent and 27 kg of cooling water/kg of solvent are applied. The amount of waste is calculated from a mass balance, and it is assumed to be sent to treatment of spent solvents since the reaction mixture contains mainly organic compounds. Excess ammonia is reported to be vented. However, if and how much of the compound that is emitted to the air is unknown, and therefore no ammonia emission is included in the unit process. Cooling water is sent to wastewater treatment. The unit-process table for this process is shown in Table S3.

$$CF_3SO_2F + NH_3 + 2\ NaOCH_3 \longrightarrow CF_3SO_2NHNa + \text{by-products} \qquad \text{(SR2)}$$

Table S3. Unit-process table for CF_3SO_2NHNa production.

Material inputs	Normalized value	Unit	Upstream data source
CF_3SO_2F	9.4E-1	kg	Section S3.1.1
Ammonia	5.0E0	kg	market for ammonia, anhydrous, liquid - RoW
Sodium methoxide	6.6E-1	kg	market for sodium methoxide - GLO
Methanol	1.6E0	kg	market for methanol - GLO
**Energy and process inputs**			
Electricity	1.3E-3	kWh	Scenario dependent, see Table S39
Steam	7.8E0	kg	market for steam, in chemical industry - RoW
Cooling energy	5.9E0	MJ	market for cooling energy - GLO
Cooling water	1.3E-1	m^3	Water, cooling, unspecified natural origin*
**Facility inputs**			
Chemical factory	4.0E-10	item	market for chemical factory, organics - GLO
**Product output**			
CF_3SO_2NHNa	1.0E0	kg	-
**Waste outputs**			
Organic waste	2.3E0**	kg	market for spent solvent mixture - RoW
Wastewater	1.3E-1	m^3	market for wastewater, average - RoW

*An elementary flow and not a process.
** Mass balance is not adding up, see explanation in the text above.

S3.1.3 $(Me_3Si)_2NH$ production

Hexamethyldisilazane (HMDS, $(Me_3Si)_2NH$) is reacted with the product from SR2, CF_3SO_2NHNa, to produce $CF_3SO_2NNaSiMe_3$ as shown in SR4. Since HMDS does not exist in the ecoinvent database or other available sources, its production is modeled in this study. Trimetylsilyl chloride and ammonia are used as reactants to produce HMDS,[16] see SR3. Inputs and outputs are obtained using stoichiometry, assuming a yield of 95%.[14] It is furthermore reported that the reaction is best performed using warm HMDS as a solvent,[16] for which the solvent amount is estimated, assuming that reactant and solvent amounts (in kg) are the same. Heating and filtration are reported process steps, where energy requirements are estimated using Eq. S1 regarding heating and 0.0055 kWh/kg dry material for filtration, both from

Piccinno et al. [9]. Since no reaction time and temperature are provided, these are assumed to be 1 hour and 100ºC, respectively. The solvent part of HMDS is assumed to be recycled at a rate of 68%.[9] Input values stated in Table S4 account for both 1 kg HMDS as product and HMDS functioning as solvent in the reaction. To obtain the amount of waste, which is assumed to be sent to spent solvent treatment due to its high content of organic compounds, mass balance calculations are performed. There is no indication that ammonium chloride formed is a co-product of this synthesis, and it is therefore assumed to become waste. The cooling water is sent to wastewater treatment. The unit-process table for this process is presented in Table S4.

$$2\ Me_3SiCl + 3NH_3 \longrightarrow (Me_3Si)_2NH + 2NH_4Cl \qquad \text{(SR3)}$$

Table S4. Unit-process table for $(Me_3Si)_2NH$ production.

Material inputs	Normalized to unit process	Unit	Upstream data source
Trimetylsilyl chloride	2.6E0	kg	dimethyldichlorosilane production - GLO*
Ammonia	6.4E-1	kg	market for ammonia, anhydrous - GLO
Energy and process inputs			
Electricity	3.6E-3	kWh	Scenario dependent, see Table S39
Steam	5.4E0	kg	market for steam, in chemical industry - RoW
Cooling water	9.5E-2	m^3	Water, cooling, unspecified natural origin**
Facility inputs			
Chemical factory	4.0E-10	Items	market for chemical factory, organics - GLO
Product output			
HMDS	1.0E0	kg	-
Waste outputs			
Organic waste	2.2E0	kg	market for spent solvent mixture - RoW
Wastewater	9.5E-2	m^3	market for wastewater, average - RoW

*Approximation of the Ecoinvent process “dimethyldichlorosilane production | dimethyldichlorosilane | Cutoff, U” based on Wickerts et al. [17].
**An elementary flow and not a process.

S3.1.4 $CF_3SO_2NNaSiMe_3$ production

When producing $CF_3SO_2NNaSiMe_3$, CF_3SO_2NHNa is reacted with HMDS, which is added in excess. Amounts of all inputs are reported, including a yield of 92%.[12] Processes such as refluxing, mixing, distillation, and drying are also mentioned. Refluxing is approximated as a distillation process, for which the same proxy values as mentioned in Section S2 are used. The energy requirement for mixing is calculated from Eq. S2, assuming a mixing time of 1 hour. Energy and cooling water inputs for distillation were approximated using the default values mentioned in Section S2, and unreacted HMDS is assumed to be recycled at a rate of 68% based on Piccinno et al. [9]. Regarding drying, for which the energy requirement is obtained using Eq. S3, there is no information about the liquid mass and exact composition of the mixture leaving the distillation and entering the drying oven. Thus, we approximate this process step by assuming that 10% of the liquid mass, which should consist mostly of HMDS, remains when the mixture is entering the oven. Again, a mass balance is used to calculate the amount of waste and the waste is assumed to be sent to treatment of spent solvents considering its high content of organic compounds. Cooling water is sent to wastewater treatment. The unit-process table for this process is presented in Table S5.

$$CF_3SO_2NHNa + (Me_3Si)_2NH \longrightarrow CF_3SO_2NNaSiMe_3 + \text{by-products} \qquad \text{(SR4)}$$

Table S5. Unit-process table for $CF_3SO_2NNaSiMe_3$ production.

Material inputs	Normalized to unit process	Unit	Upstream data source
CF_3SO_2NHNa	7.6E-1	kg	Section S3.1.2
$(Me_3Si)_2NH$	2.2E0	kg	Section S3.1.3
Energy and process inputs			
Electricity	2.3E-2	kWh	Scenario dependent, see Table S39
Steam	1.6E1	kg	market for steam, in chemical industry - GLO
Cooling water	2.8E-1	m^3	Water, cooling, unspecified natural origin*
Facility inputs			
Chemical factory	4.0E-10	items	market for chemical factory, organics - GLO
Product output			
$CF_3SO_2NNaSiMe_3$	1.0E0	kg	-
Waste outputs			
Organic waste	2.0E0	kg	market for spent solvent mixture - RoW
Wastewater	2.8E-1	m^3	market for wastewater, average - RoW

*An elementary flow and not a process.

S3.1.5 $(CF_3SO_2)_2NNa$ production

$(CF_3SO_2)_2NNa$ is synthesized using $CF_3SO_2NNaSiMe_3$ and trifluoromethanesulfonyl fluoride in tetrahydrofuran as starting materials, see SR5. Input amounts and a yield of 98% are provided.[12] Stirring of the reaction mixture at 100ºC overnight is reported, thus a reaction time of 12 hours is assumed, and the corresponding energy requirement is calculated using Eq. S1 and S2. Water and dichloromethane are then added, for which the amount of water is given while the dichloromethane amount is approximated. Rotary evaporation is used to remove the solvents, which is again approximated as a distillation with 1.5 kg steam and 27 kg of cooling water per kg of solution. A drying step is mentioned, and again there is no information regarding the dried liquid mass, which is why 10% of the original liquid mass is assumed. The composition of the mixture entering the drying oven is unknown as well. Hence, a simplification is made by assuming the same liquid composition as that of the added input materials (22 wt% tetrahydrofuran, 34 wt% water and 44 wt% dichloromethane). The amount of waste is obtained from the mass balance of the entire process and includes reaction by-

products. Considering the organic constituents of the solution, the waste is assumed to be sent to spent solvent treatment, while the cooling water is sent to wastewater treatment. The unit-process table for this process is presented in Table S6.

$CF_3SO_2NNaSiMe_3 + CF_3SO_2F \longrightarrow (CF_3SO_2)_2NNa$ + by-products (SR5)

Table S6. Unit-process table for $(CF_3SO_2)_2NNa$ production.

Material inputs	Normalized to unit process	Unit	Upstream data source
$CF_3SO_2NNaSiMe_3$	8.2E-1	kg	Section S3.1.4
CF_3SO_2F	5.2E-1	kg	Section S3.1.1
Tetrahydrofuran	6.5E-1	kg	market for tetrahydrofuran - GLO
Dichloromethane	1.3E0	kg	market for dichloromethane - RoW
Water, deionized	3.1E0	kg	market for water, deionised - RoW
Energy and process inputs			
Electricity	4.4E-1	kWh	Scenario dependent, see Table S39
Steam	2.6E1	kg	market for steam, in chemical industry - RoW
Cooling water	3.8E-1	m^3	Water, cooling, unspecified natural origin*
Facility inputs			
Chemical factory	4.0E-10	items	market for chemical factory, organics - GLO
Product output			
$(CF_3SO_2)_2NNa$	1.0E0	kg	-
Waste outputs			
Organic waste	5.4E0	kg	market for spent solvent mixture - RoW
Wastewater	3.8E-1	m^3	market for wastewater, average - RoW

*An elementary flow and not a process.

S3.1.6 $(CF_3SO_2)_2NH$ production

The next step is to let $(CF_3SO_2)_2NNa$ react with concentrated sulfuric acid to produce bis(trifluoromethane)sulfonimide ($(CF_3SO_2)_2NH$),[12] see SR6. Input and output amounts are provided, and the reaction yield is reported to be 93%. Heating of the reaction mixture is reported, for which the energy requirement is obtained by using Eq. S1. The amount of waste is obtained by mass balance calculations and the waste is assumed to be sent to wastewater

treatment due to the use of an aqueous solvent (sulfuric acid). No by-products are mentioned in the laboratory description, but to the extent such are produced in the reaction, they are assumed to become liquid waste as well and follow the spent solvent and unreacted reactants to the wastewater treatment. The unit-process table for this process is presented in Table S7.

$(CF_3SO_2)_2NNa + H_2SO_4 \longrightarrow (CF_3SO_2)_2NH + \text{by-product}$ (SR6)

Table S7. Unit-process table for $(CF_3SO_2)_2NH$ production.

Material inputs	Normalized to unit process	Unit	Upstream data source
$(CF_3SO_2)_2NNa$	1.2E0	kg	Section S3.1.5
Sulfuric acid	2.5E0	kg	market for sulfuric acid - RoW
**Energy and process inputs**			
Steam	1.2E-1	kg	market for steam, in chemical industry - RoW
**Facility inputs**			
Chemical factory	4.0E-10	items	market for chemical factory, organics - GLO
**Product output**			
$(CF_3SO_2)_2NH$	1.0E0	kg	-
**Waste outputs**			
Wastewater	2.7E0	kg	market for wastewater, average - RoW

S3.1.7 $(CF_3SO_2)_2NLi$ production

LiTFSI ($(CF_3SO_2)_2NLi$) is produced by reacting $(CF_3SO_2)_2NH$ with lithium carbonate (Li_2CO_3),[18] see SR7. For this reaction, input and output amounts are obtained from stoichiometry, with the inputs adjusted by a reaction yield of 66% based on Friedrich et al. [18]. Mixing, filtration, and drying are reported by the authors. Corresponding energy requirements for these processes are obtained using Eq. S2 and S3 regarding mixing and drying, respectively, and 0.0055 kWh/kg dry material for filtration. Regarding co-products, it can be seen in SR7 that lithium bicarbonate is produced. Contrary to all previous by-products in the LiTFSI production system, the lithium bicarbonate is likely to become utilized within the process in the future, considering that it can relatively easily be used to produce the lithium carbonate input. The patent by Perez et al. [19] is used for modeling the conversion of lithium bicarbonate back to lithium carbonate. As shown in SR8, lithium bicarbonate decomposes to lithium

carbonate when subjected to heat. First, lithium bicarbonate is dissolved in water. The reaction mixture is then filtered to remove impurities, which is followed by a heating step to produce lithium carbonate. Filtration, washing the lithium carbonate filter cake with water and drying are reported to yield battery-grade lithium carbonate, for which the corresponding energy requirements are obtained using the drying equation (Eq. S3) and the filtration value (0.0055 kWh/kg) previously mentioned. The produced lithium carbonate is assumed to be used in the LiTFSI production and thus reduces the required net input amount of lithium carbonate. The amount of waste generated is calculated from the mass balance, and is assumed to be sent to wastewater treatment since water is used as solvent. It shall be noted that the amount of wastewater is uncertain, as there is a drying step involved (with no information of how much of the water is evaporated). Carbon dioxide is produced in SR8 and is assumed to become emitted to air. The unit-process table for this process is presented in Table S8.

$(CF_3SO_2)_2NH + Li_2CO_3 \longrightarrow (CF_3SO_2)_2NLi + LiHCO_3$ (SR7)

$2\ LiHCO_3 + \text{heat} \longrightarrow Li_2CO_3 + H_2O + CO_2$ (SR8)

Table S8. Unit-process table for LiTFSI production.

Material inputs	Normalized to unit process	Unit	Upstream data source
$(CF_3SO_2)_2NH$	1.5E0	kg	Section S3.1.6
Lithium carbonate	2.7E-1	kg	market for lithium carbonate | lithium carbonate*
Water, deionized	1.5E1	kg	market for water, deionised - RoW
Energy and process inputs			
Electricity	5.5E0	kWh	Scenario dependent, see Table S39
Steam	2.4	kg	market for steam, in chemical industry - RoW
Facility inputs			
Chemical factory	4.0E-10	items	market for chemical factory, organics - GLO
Product output			
LiTFSI	1.0E0	kg	-
Process emissions			
Carbon dioxide	7.7E-2	kg	-
Waste outputs			
Wastewater	1.6E1	kg	market for wastewater, average - RoW

*Market process adjusted to only contain primary lithium carbonate, since a closed-loop recycling of lithium carbonate is modeled in this study. See further the "Materials and Methods" section in the main manuscript.

S3.2 Lithium triflate production

The modeling of lithium triflate (LiOTf) is based on reacting triflic acid (CF_3SO_3H) with lithium carbonate.[18] Production of LiOTf involves several synthesis steps, which are shown in the flow chart in Figure S2.

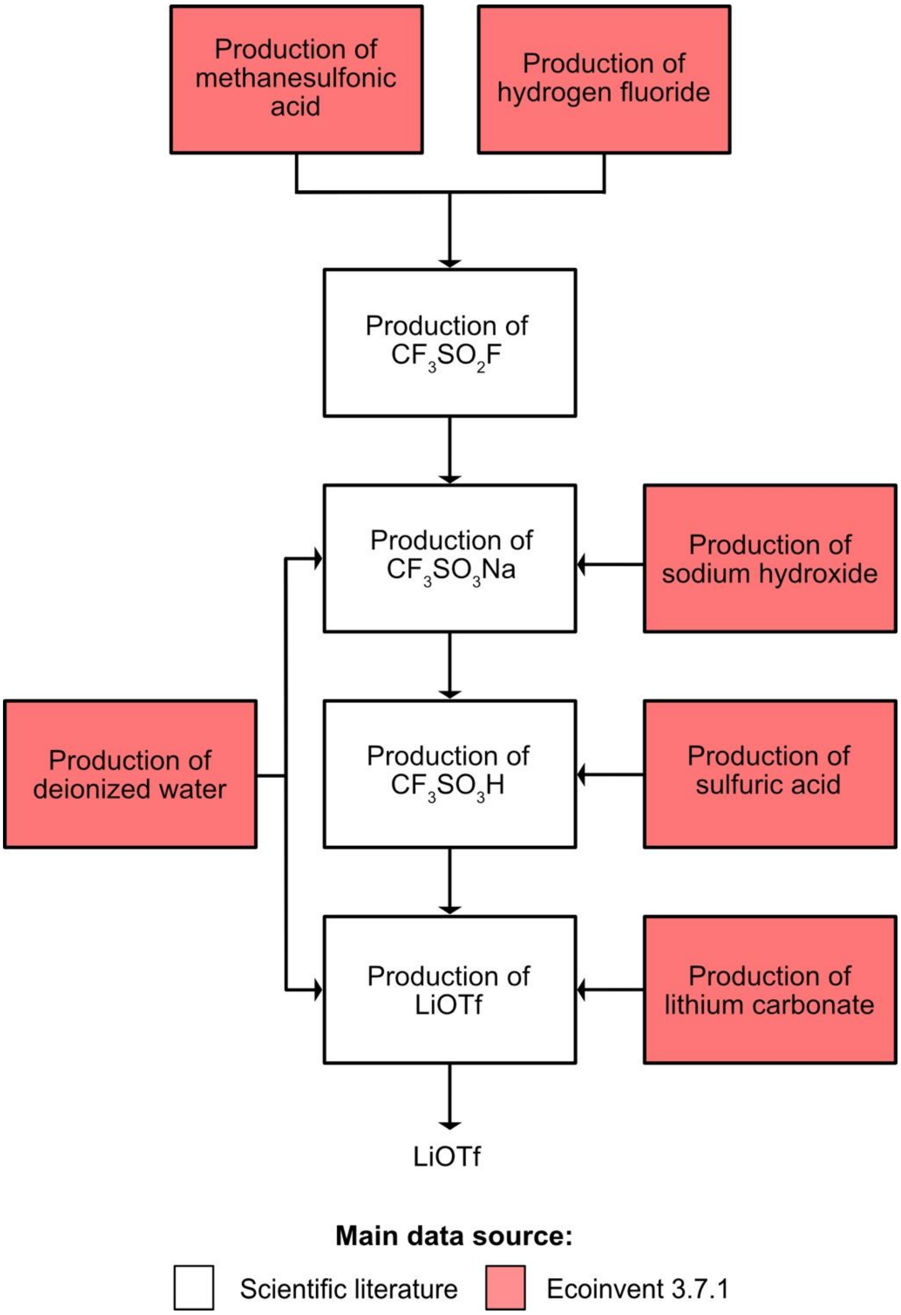

Figure S2. Flowchart of LiOTf production.

S3.2.1 CF_3SO_3Na and CF_3SO_3H production

As for LiTFSI, CF_3SO_2F is a required precursor for LiOTf and is produced from electrochemical fluorination of methanesulfonic acid, see SR1, for which the same modeling is applied. Alkaline hydrolysis of CF_3SO_2F gives the salt CF_3SO_3Na, as can be seen in SR9.[13] The salt is in turn reacted with concentrated sulfuric acid and distilled, which yields triflic acid, see SR10.

$$CF_3SO_2F + NaOH \longrightarrow CF_3SO_3Na + HF \qquad \text{(SR9)}$$

$CF_3SO_3Na + H_2SO_4 \longrightarrow CF_3SO_3H + NaHSO_4$ (SR10)

Based on the chemical reactions SR9 and SR10, inputs and outputs are obtained by stoichiometric calculations. Since no reaction yield is reported, 95% is assumed.[14] Due to lack of information, a reaction time of one hour and a reaction temperature of 100ºC are assumed, and Eq. S1 is used to obtain the energy requirement from the heating. Apart from heating, stirring is also assumed to be necessary, for which the energy requirement is calculated using Eq. S2. Distillation is mentioned for the production of triflic acid, and the same proxy values as in Section S2 are used for steam and cooling water. The amount of waste is calculated from a mass balance and is assumed to be sent to wastewater treatment due to the use of water as solvent, as well as the inorganic chemicals sodium hydroxide and sulfuric acid. The unit-process tables for CF_3SO_3Na and triflic acid production are provided in Table S9 and S10, respectively.

Table S9. Unit-process table for CF_3SO_3Na production.

Material inputs	Normalized to unit process	Unit	Upstream data source
CF_3SO_2F	9.3E-1	kg	Section S3.1.1
Sodium hydroxide	2.4E-1	kg	market for sodium hydroxide, without water, in 50% solution state - GLO
Water, deionized	2.4E0	kg	market for water, deionised - RoW
Energy and process inputs			
Electricity	4.2E-5	kWh	Scenario dependent, see Table S39
Steam	4.1E-1	kg	market for steam, in chemical industry - RoW
Facility inputs			
Chemical factory	4.0E-10	item	market for chemical factory, organics - GLO
Product output			
CF_3SO_3Na	1.0E0	kg	-
Waste outputs			
Wastewater	2.5E0	kg	market for wastewater, average - RoW

Table S10. Unit-process table for production of triflic acid.

Material inputs	Normalized to unit process	Unit	Upstream data source
CF_3SO_3Na	1.2E0	kg	Section S3.2.1
Sulfuric acid	6.9E-1	kg	sulfuric acid production - RoW
Energy and process inputs			
Electricity	1.2E-5	kWh	Scenario dependent, see Table S39
Steam	3.0E0	kg	market for steam, in chemical industry - RoW
Cooling water	5.1E-2	m^3	Water, cooling, unspecified natural origin*
Facility inputs			
Chemical factory	4.0E-10	item	market for chemical factory, organics - GLO
Product output			
CF_3SO_3H	1.0E0	kg	-
Waste outputs			
Wastewater	5.2E-2	m^3	market for wastewater, average - RoW

*An elementary flow and not a process.

S3.2.2 $LiCF_3SO_3$ production

In the next reaction step, triflic acid is reacted with lithium carbonate, see SR11. Stoichiometry is used to obtain input and output values, and the inputs are adjusted by a reaction yield of 66% based on Friedrich et al. [18]. This reaction step follows the same process description as the last reaction step in the LiTFSI production, including mixing, filtration and drying. The energy requirements for the process steps are calculated using Eq. S2, Eq. S3 and the default value for filtration provided in Section S2. Carbonic acid in SR11 is assumed to react into water and carbon dioxide, see SR12. The stochiometric amount of carbon dioxide is modeled to be emitted to the air. The amount of waste is calculated from a mass balance and is assumed to be sent to wastewater treatment due to the use of water as solvent. The unit-process table for this process is presented in Table S11.

$$2\ CF_3SO_3H + Li_2CO_3 \longrightarrow 2\ LiCF_3SO_3 + H_2CO_3 \qquad \text{(SR11)}$$

$$H_2CO_3 \longrightarrow H_2O + CO_2 \qquad \text{(SR12)}$$

Table S11. Unit-process table for LiOTf production.

Material inputs	Normalized to unit process	Unit	Upstream data source
CF_3SO_3H	1.5E0	kg	Section S3.2.1
Lithium carbonate	3.6E-1	kg	market for lithium carbonate | lithium carbonate*
Water, deionized	8.9E0	kg	market for water, deionised - RoW
Energy and process inputs			
Electricity	5.1E0	kWh	Scenario dependent, see Table S39
Facility inputs			
Chemical factory	4.0E-10	items	market for chemical factory, organics - GLO
Product output			
LiOTf	1.0E0	kg	-
Process emissions			
Carbon dioxide	2.8E-1	kg	-
Waste outputs			
Wastewater	9.5E0	kg	market for wastewater, average - RoW

*Market process adjusted to only contain primary lithium carbonate, since a closed-loop recycling of lithium carbonate is modeled in this study. See further Section 2.4 in the main manuscript.

S3.3 Polyehtylene glycol production

Polyethylene glycol (PEG) is modeled based on a patent by Daugs et al. [20], for which the flow chart is presented in Figure S3. The polymer is produced by reacting diethylene glycol dimethyl ether, also called diglyme, with ethylene oxide, see SR13. The reaction temperature is 100ºC and ethylene oxide is continuously added for seven hours. The weight percentage of PEG in the product mixture after the reaction is reported to be just over 50%, implying a 50% yield.

$$C_6H_{14}O_3 + C_2H_4O \longrightarrow C_{2n}H4_{n+2}O_{n+1} \qquad \text{(SR13)}$$

Inputs amounts are given in the patent. Production of the diglyme input is approximated using the ecoinvent market process for ethylene glycol dimethyl ether, since both chemicals are based on the same starting materials. In addition, anhydrous diethylene glycol methyl ether is reported to be used as an auxiliary chemical. However, due to the small amount required (~4 g/kg product), it is not included in the modeling. Energy requirements for heating is calculated using Eq. S1. The amount of waste is calculated based on a mass balance calculation, which is

assumed to be sent to spent solvent treatment for incineration due to the reaction mixture's organic composition. The unit-process table for this process is presented in Table S12.

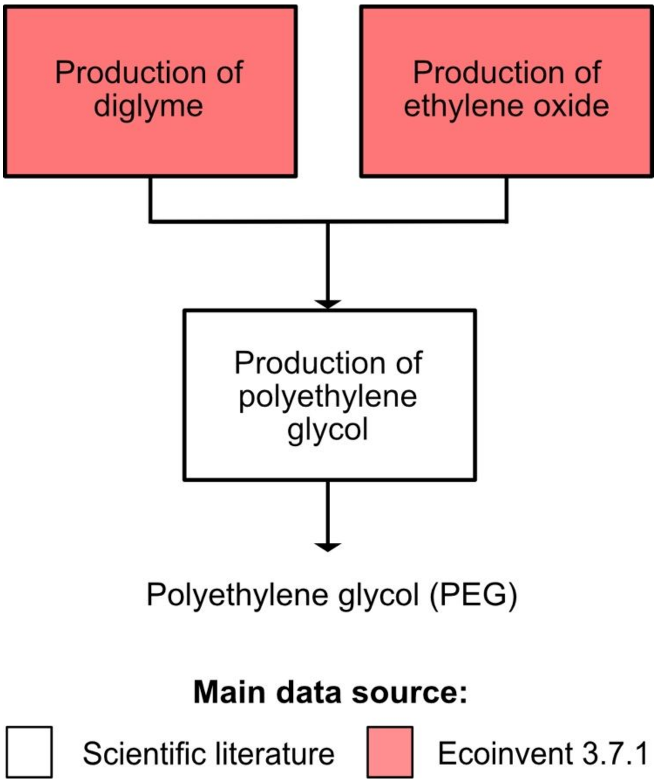

Figure S3. Flowchart for PEG production.

Table S12. Unit-process table for PEG production.

Material inputs	Normalized to unit process	Unit	Upstream data source
Diglyme	9.8E-1	kg	market for ethylene glycol dimethyl ether - GLO
Ethylene oxide	1.0E0	kg	market for ethylene oxide - RoW
Energy and process inputs			
Steam	1.2E-1	kg	market for steam, in chemical industry - RoW
Facility inputs			
Chemical factory	4.0E-10	items	market for chemical factory, organics - GLO
Product output			
PEG	1.0E0	kg	-
Waste outputs			
Organic waste	9.8E-1	kg	market for spent solvent mixture - RoW

S3.4 Sulfolane production

Sulfolane ($(CH_2)_4SO_2$) is produced via hydrogenation of sulfolene ($(C_4H_6)SO_2$), which in turn is synthesized by reacting 1,3-butadiene with sulfur dioxide, according to the patent by Clark Jr. and Straw [21]. The flowchart is presented in Figure S4 and the chemical reactions in SR14 and SR15, respectively.

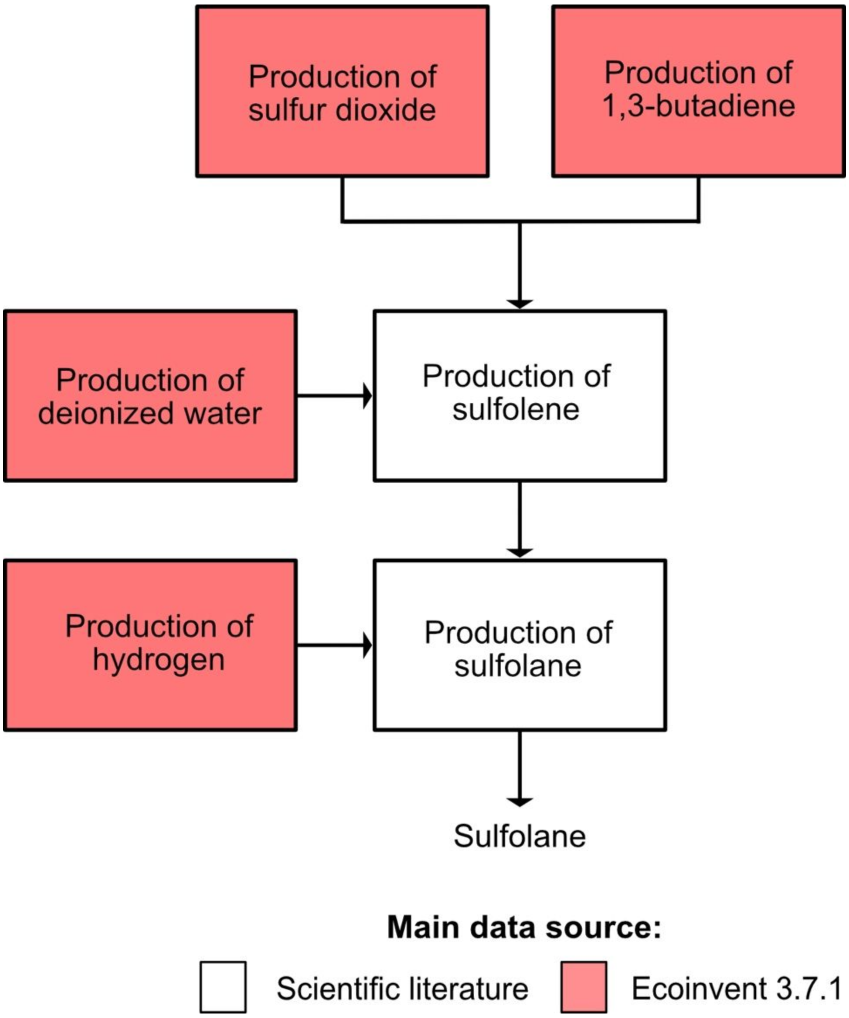

Figure S4. Flowchart of sulfolane production.

Input values for the sulfolene synthesis are provided in the patent, and so is the sulfolane output. Also, the mass of the auxiliary materials dimethylamine and water are given. However, since the dimethylamine amount is small (~3 g/kg product), it is not included in the modeling. Furthermore, sulfolene is also used as a solvent in this first reaction step, which is assumed to be recovered by distillation. The authors indicate that a large part of the solvent can be recycled. Hence, a recovery rate of 95% is assumed in this case. Therefore, the sulfolene reactant inputs (i.e., sulfur dioxide and 1,3-butadiene) are set to stoichiometrically match the unrecovered sulfolene, as well as the stoichiometrically amounts required for the actual reaction (SR14).

Heating and mixing are reported by the authors, for which the energy requirements are calculated using Eq. S1 and S2. Since Clark Jr. and Straw [21] report heating by an electric heater, the required energy is not converted into steam in this case but modeled as electricity.

$SO_2 + C_4H_6 \longrightarrow C_4H_6O_2S$ (SR14)

$C_4H_6O_2S + H_2 \longrightarrow C_4H_8O_2S$ (SR15)

Hydrogenation of sulfolene requires a Raney nickel catalyst (~75 g/kg product).[21] The catalyst is, however, assumed to be recycled efficiently, and is therefore excluded in this modeling. Since the sulfolane output is given in the patent, the hydrogen input can be stoichiometrically calculated, assuming a 95% yield.[14] The hydrogenation reaction is exothermic, but cooling water is reported to be used to maintain a reaction temperature of 50ºC. However, since there is no information about how much cooling water is required, it is excluded from the modeling. The amount of waste is calculated from a mass balance. The waste is assumed to be sent to spent solvent treatment, even though there are both organic and inorganic chemicals in the reaction mixture. The cooling water used in the distillation process, however, is assumed to be sent to wastewater treatment. The unit-process table for this process is presented in Table S13.

Table S13. Unit-process table for sulfolane production.

Material inputs	Normalized to unit process	Unit	Upstream data source
1,3-butadiene	7.4E-1	kg	butadiene production - RoW
Hydrogen, gaseous	2.1E-2	kg	hydrogen production, gaseous, petroleum refinery operation - RoW
Sulfur dioxide	9.2E-1	kg	market for sulfur dioxide, liquid - RoW
Water, deionized	6.3E-1	kg	market for water, deionised - RoW
Energy and process inputs			
Electricity	1.3E-1	kWh	Scenario dependent, see Table S39
Steam	6.5E0	kg	market for steam, in chemical industry - RoW
Cooling water	1.2E-1	m^3	Water, cooling, unspecified natural origin*
Facility inputs			
Chemical factory	4.0E-10	item	market for chemical factory, organics -GLO
Product output			
Sulfolane	1.0E0	kg	-
Waste outputs			
Organic waste	1.3E0	kg	market for spent solvent mixture - RoW
Wastewater	1.2E-1	m^3	market for wastewater, average - RoW

*An elementary flow and not a process.

S3.5 Mesoporous carbon production

CMK-3 is a mesoporous carbon, for which the production is modeled based on Jun et al. [22]; see flowchart in Figure S5. The mesoporous carbon is synthesized by impregnating a silica template, specifically SBA-15, with sulfuric acid and sucrose, where the latter functions as the carbon source. To produce SBA-15, the co-polymer pluronic P123 is required.

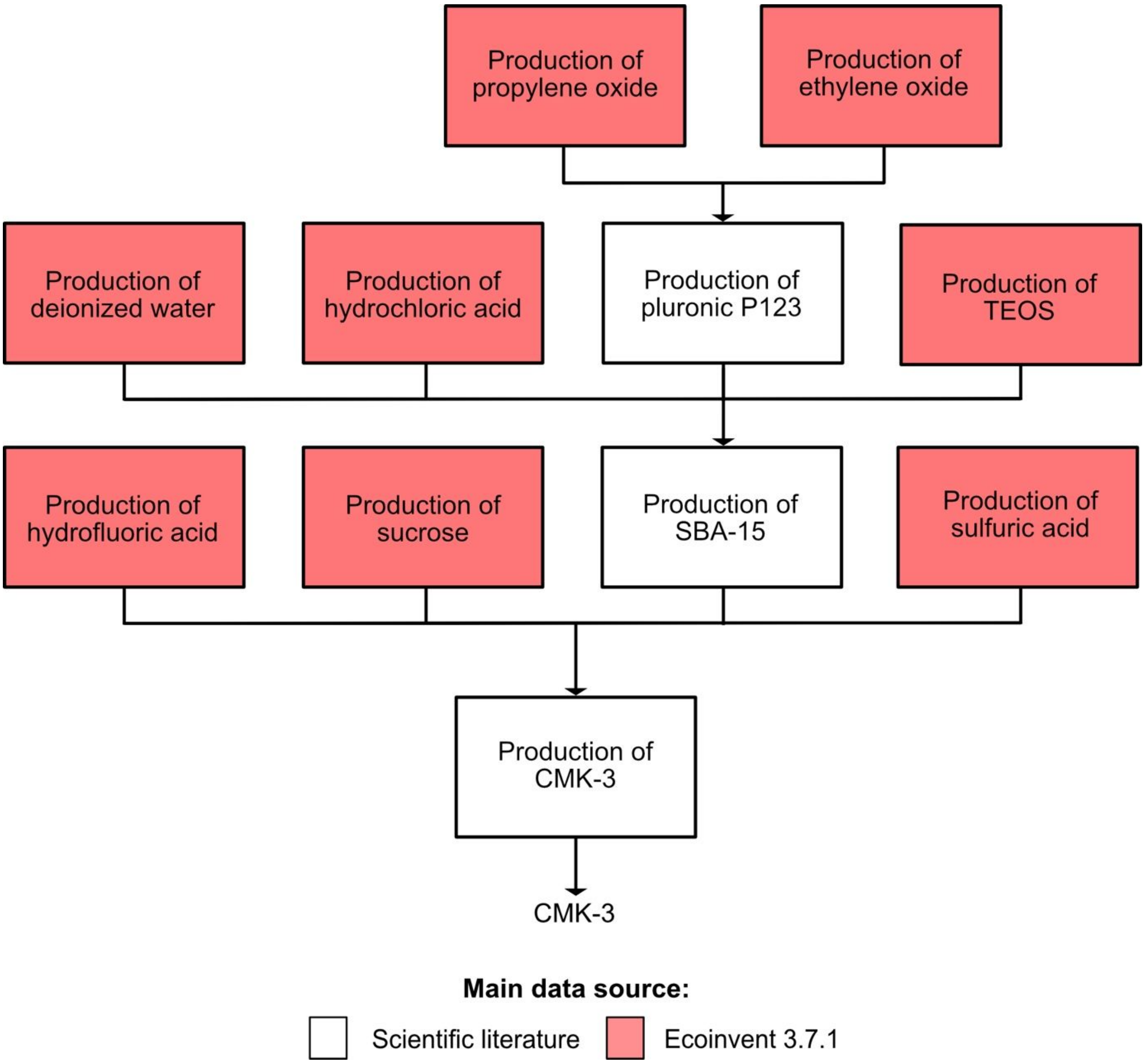

Figure S5. Flowchart of CMK-3 production.

S3.5.1 Pluronic P123 production

Pluronic P123 is a co-polymer, which consists of blocks of polyethylene (PE) and polypropylene (PP). Ethylene oxide and propylene oxide are the inputs to the polymerization reaction, which are calculated for 1 kg of polymer with a 70/40 molar ratio of ethylene/propylene, respectively.[23] As no yield could be found, 95% is used.[14] The general polymerization reaction presented in Moulijn et al. [24] is the basis for calculating the energy requirements of the different process steps, where an autoclave with stirring equipment is used. The residence time is between 30 and 60 seconds,[24] and a reaction temperature of 120°C is reported for this type of co-polymer in Noshay and McGrath [25] and therefore applied here. Consequently, heating and stirring are modeled using Eq. S1 and S2, respectively, with a reaction temperature of 120°C and a reaction time of 45 seconds. The amount of waste is again

calculated based on a mass balance, which is assumed to be sent to spent solvent treatment for incineration due to the organic constituents of the reaction mixture. The unit-process table for this process is presented in Table S14.

Table S14. Unit-process table for production of Pluronic P123.

Inputs	Normalized to unit process	Unit	Upstream data source
Ethylene oxide	3.2E-1	kg	market for ethylene oxide - RoW
Propylene oxide	7.4E-1	kg	propylene oxide production, liquid - RoW
Process inputs			
Electricity	2.1E-7	kWh	Scenario dependent, see Table S39
Steam	1.1E-1	kg	market for steam, in chemical industry - RoW
Facility inputs			
Chemical factory	4.0E-10	items	market for chemical factory, organics - GLO
Product output			
Pluronic P123	1.0E0	kg	-
Waste outputs			
Organic waste	6.0E-2	kg	market for spent solvent mixture - RoW

S3.5.2 SBA-15 production

The production of SBA-15 is also based on Jun et al. [22]. SBA-15 is synthesized by letting the co-polymer Pluronic P123 react with a silica source, here tetraethyl orthosilicate (TEOS). Hydrochloric acid is also added, and water functions as solvent, see the flowchart in Figure S5. The inputs for 1 g of SBA-15 are provided in the experimental study. Stirring and drying with specific reaction temperatures and reaction times are reported by the authors, and filtration is also mentioned. The corresponding energy requirements are obtained using Eq. S2 and S3 for the first two, and 0.0055 kWh of electricity per kg dry material for the filtration. It is assumed that all liquid will be removed during drying. While waste might be generated during the production process, it is neglected due the uncertainty of its amount. The unit-process table for this process is presented in Table S15.

Table S15. Unit-process table for production of SBA-15.

Inputs	Normalized to unit process	Unit	Upstream data source
Pluronic P123	5.2E-1	kg	Section S3.5.1
TEOS	1.1E0	kg	market for tetraethyl orthosilicate - GLO
Hydrochloric acid	1.1E0	kg	market for hydrochloric acid, without water, in 30% solution state |- RoW
Water, deionized	1.8E1	kg	market for water, deionised - RoW
Process inputs			
Electricity	1.7E1	kWh	Scenario dependent, see Table S39
Facility inputs			
Chemical factory	4E-10	items	market for chemical factory, organics - GLO
Product output			
SBA-15	1.0E0	kg	-

S3.5.3 CMK-3 production

For CMK-3 production, input amounts per 1 g of output is provided in Jun et al. [22]. Drying is reported on several occasions throughout the synthesis, for which the energy requirement is obtained using Eq. S3. It is assumed that all liquid is removed during drying. While waste might be generated during the production process, it is neglected due the uncertainty of its amount. The unit-process table for this process is presented in Table S16.

Table S16. Unit-process table for production of CMK-3.

Material inputs	Normalized to unit process	Unit	Upstream data source
SBA-15	1.0E0	kg	Section S3.5.2
Sucrose	2.1E0	kg	market for sugar, from sugar beet - GLO
Sulfuric acid	2.3E-1	kg	market for sulfuric acid - RoW
Water, deionized	1.0E1	kg	market for water, deionised - RoW
Hydrogen fluoride	1.0E0	kg	market for hydrogen fluoride - RoW
Energy and process inputs			
Electricity	2.4E1	kWh	Scenario dependent, see Table S39
Facility inputs			
Chemical factory	4.0E-10	items	market for chemical factory, organics - GLO
Product output			
CMK-3	1.0E0	kg	-

S3.6 Cell production

The cell production is mainly modeled based on Chordia et al. [26], who model large-scale ("gigafactory") production of lithium-ion battery cells, specifically the nickel-manganese-cobalt chemistry with an 8:1:1 proportion (NMC811) and a cylindrical geometry. However, considering the differences between the Li-S cell in this study and the NMC811 cell, some processes are modified as described below. A process flow chart of Li-S cell production is provided in Figure S6.

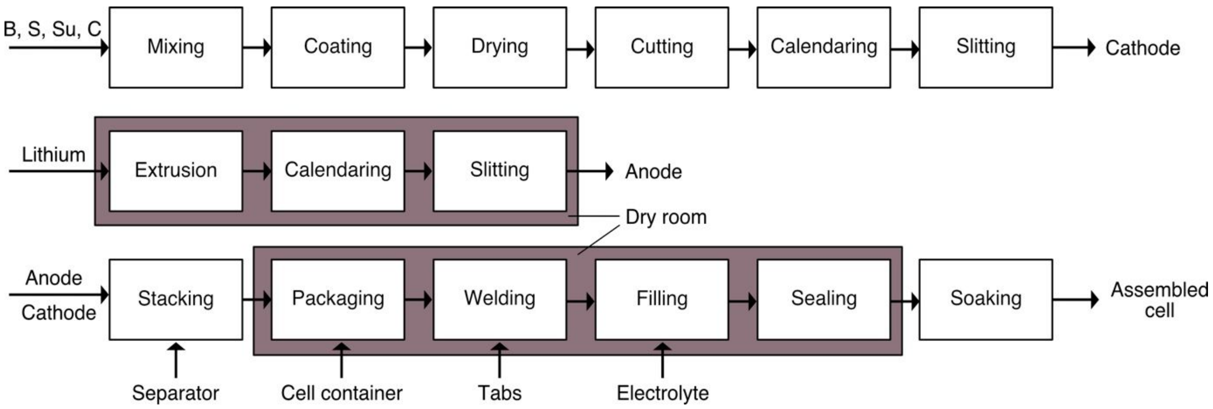

Figure S6. Processes included in the cell production: cathode production, anode production and cell assembly. Flowcharts for the cathode and cell assembly are based on Chordia et al. [27] and Jinasena et al. [28], whereas the anode flowchart is based on several sources (see Section S3.6.1). B=binder, S=solvent, Su=sulfur, C=carbon material.

S3.6.1 Lithium foil production

Production of the lithium anode foil is mostly based on other sources than Chordia et al. [27], since that study considers a graphite anode for the NMC811 cell. Lithium foil is produced by first extruding liquid metallic lithium into a foil, which is then subject to calendaring to ensure foil thickness and homogeneity.[1, 29] The foil is then slitted into smaller sections,[30] for which the energy consumption is approximated based on the gigafactory model.[27] The exact values are obtained from personal communication with the first author of Chordia et al. [27]. The electricity consumption for lithium extrusion is based on Deng et al. [1]. Regarding calendaring, the ecoinvent process "sheet rolling, aluminum" is used as a proxy. Since aluminum and lithium have different densities, an adjustment factor of 4.95 based on Deng et al. [1] is applied to the sheet rolling process. In Deng et al. [1], there is a 4% lithium loss modeled. However, in this study, the generated lithium waste is calculated from a mass balance, and while Deng et al. [1] landfills this waste, we assume that it can be recycled back into the process and thus do not assume any losses. Furthermore, due to lithium's reactivity, an inert or dry environment is required,[30] which is why the lithium foil production is modeled in a dry room. Energy requirements for the dry room are retrieved from personal communication with the first author of Chordia et al. [27]. We assume the anode is produced in the same facility as the other battery cell components, thus no additional requirements for factory construction and operation are added. The unit-process table for this process is presented in Table S17 and the flowchart in Figure S7.

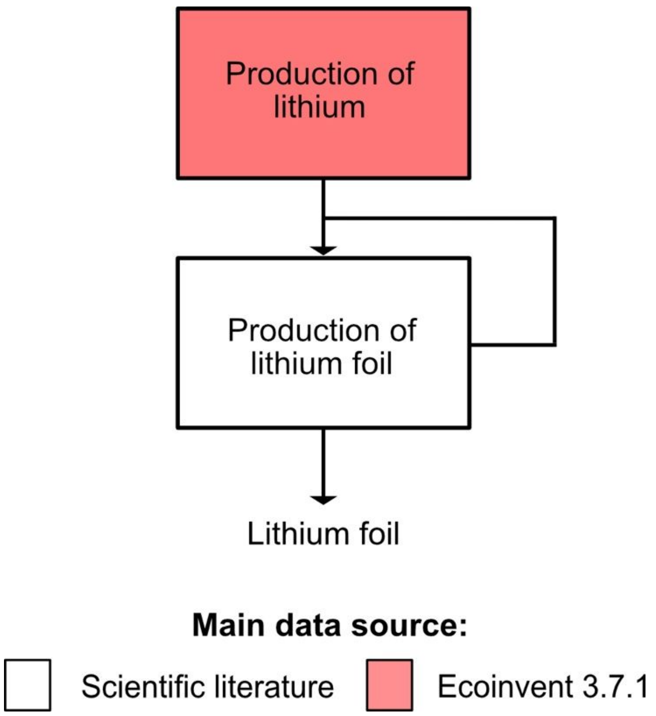

Figure S7. Flowchart of lithium foil production, where foil production losses of lithium are assumed to be recycled back into the lithium foil production process.

Table S17. Unit-process table for production of the lithium foil.

Material inputs	Normalized to unit process	Unit	Upstream data source
Lithium	1.00E0	kg	electrolysis of lithium chloride - GLO
Energy and process inputs			
Sheet rolling	4.95E0	kg	sheet rolling, aluminium - RoW*
Electricity	2.10E0	kWh	Scenario dependent, see Table S39
District heating	5.00E0	MJ	market for heat, district or industrial, natural gas - RoW
District cooling	4.20E0	MJ	market for cooling energy - GLO
Product output			
Lithium foil	1.00E0	kg	-

*A lithium input is used instead of an aluminum input and the required amount of sheet rolling is adjusted based on the lower density of lithium.

3.6.2 Separator production

The production of the separator is modeled based on Li et al. [31]. However, that separator contains PE only, while the separator in this study contains both PE and PP. Therefore, the

inputs in the unit process provided in Li et al. [31] are modified to contain both PE and PP, and the ratio between the two is based on the separator modeled in Deng et al. [1]. Waste amounts are calculated from a mass balance and the waste is assumed to be sent to spent solvent treatment due to the organic compounds in the reaction mixture. The unit-process table for this process is presented in Table S18 and the flowchart in Figure S8.

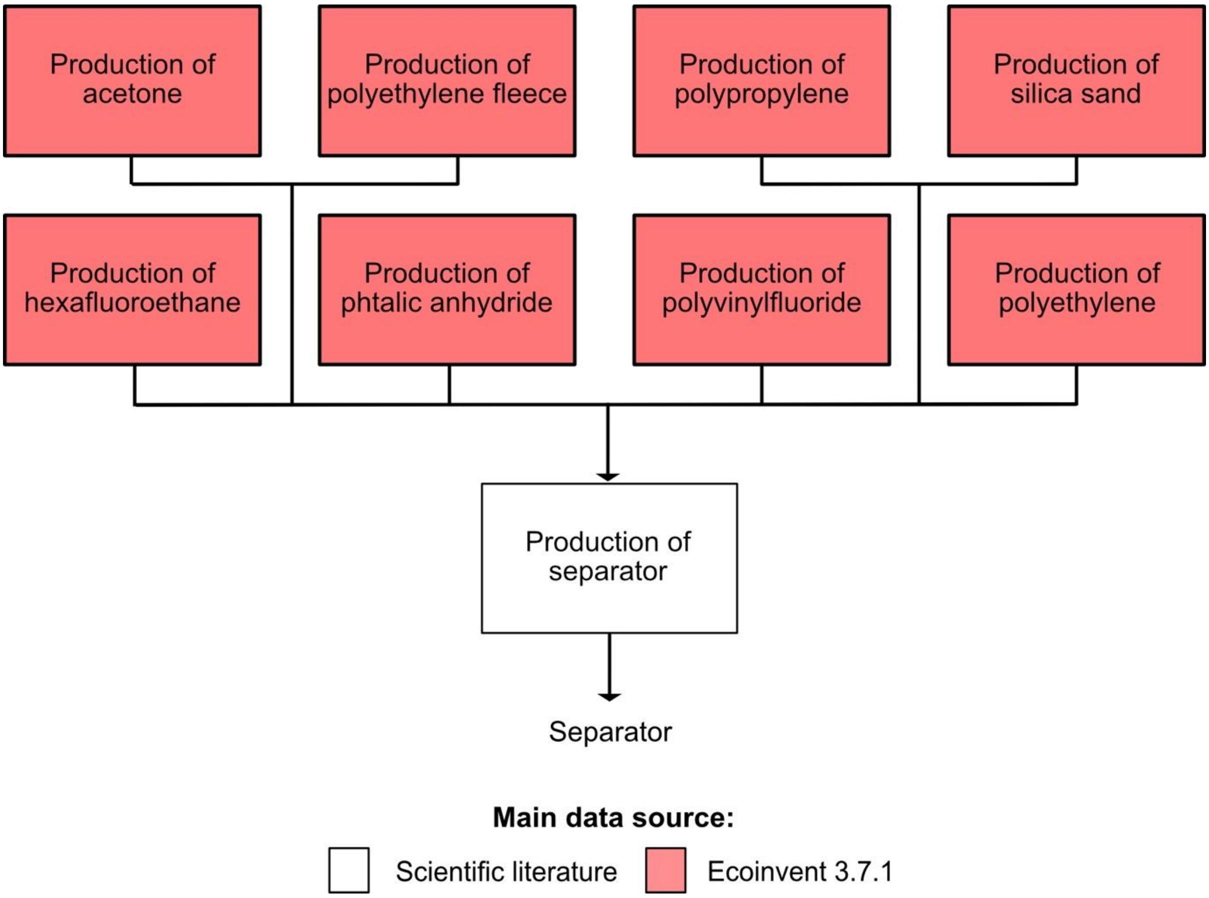

Figure S8. Flowchart of separator production.

Table S18. Unit-process table for production of the separator.

Material inputs	Normalized to unit process	Unit	Upstream data source
Acetone	1.4E-1	kg	market for acetone, liquid - RoW
Fleece, polyethylene	1.2E-1	kg	market for fleece, polyethylene - GLO
Hexafluoroethane	2.6E-2	kg	market for hexafluoroethane - GLO
Phtalic anhydride	2.9E-1	kg	market for phthalic anhydride - GLO
Polypropylene, granulate	1.0E0	kg	market for polypropylene, granulate - GLO
Polyvinylfluoride	1.9E-1	kg	market for polyvinylfluoride - GLO
Silica sand	2.2E-1	kg	market for silica sand - GLO
Polyethylene, granulate	3.9E-1	kg	market for polyethylene, granulate - GLO
Energy and process inputs			
Electricity	5.8E-2	kWh	Scenario dependent, see Table S39
Heat	1.9E-1	MJ	heat production, natural gas, at industrial furnace >100kW
Infrastructure			
Chemical factory	4.0E-10	item	market for chemical factory, organics - GLO
Product output			
Separator	1.0E0	kg	-
Waste outputs			
Organic waste	1.4E0	kg	market for spent solvent mixture - RoW

S3.6.3 Cathode production

The composite cathode is produced by mixing elemental sulfur with a carbon material and a binder material in a solvent. Acetonitrile is used as solvent according to Kolosnitsyn and Karaseva [32]. The cathode slurry is coated onto an aluminum foil. Both sides are assumed to be coated and then the cathode is dried. After drying, the cathode is subjected to cutting, calendaring and slitting.[27] Cathode composition is based on Arvidsson et al. [2]. However, no binder is included in that study, but in this study a binder constituting 20 wt% of the cathode is assumed, following Kolosnitsyn and Karaseva [32]. In line with Arvidsson et al. [2], CMK-3 is the carbon material considered in the base scenario, energy system scenario and technical performance scenario, while carbon black is modeled in the material selection and combined scenarios in line with Kolosnitsyn and Karaseva [32]. Following Deng et al. [1], polyvinylidene

fluoride (PVDF) is the binder of choice in the base scenario, energy system scenario and technical performance scenario, while the PEG binder is used in the material selection and combined scenarios, following Kolosnitsyn and Karaseva [32]. Energy requirements for the cathode processing are obtained from Chordia et al. [27], which includes a 99% solvent recovery. Consequently, we assume a solvent input of 1% of the required amounts in the cathode processing. The waste amount is calculated from a mass balance and the waste is assumed to be sent to spent solvent treatment due to the use of the organic acetonitrile solvent. The unit-process table for this process is presented in Table S19 and the flowchart in Figure S9.

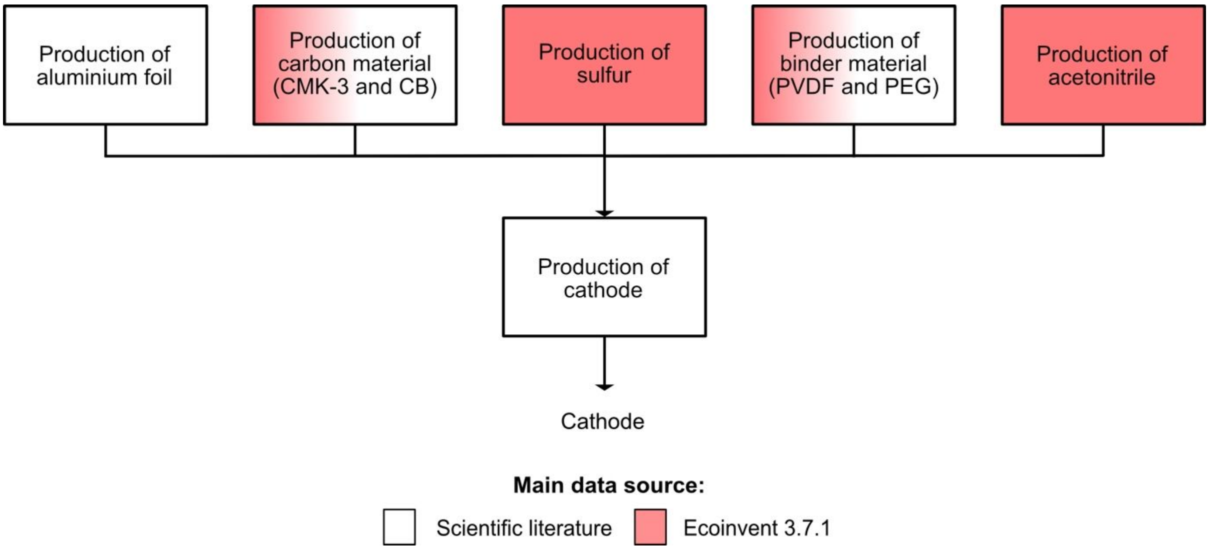

Figure S9. Flowchart of cathode production.

Table S19. Unit-process table for production of the cathode. B=base scenario, M=material selection scenario, E=energy system scenario, T=technical performance scenario, R=recycling scenario, C=combined scenario, n/a=not applicable.

Material inputs	B	M	E	T	R	C	Unit	Upstream data source
Sulfur	2.8E-1	2.8E-1	2.8E-1	6.8E-1	2.8E-1	6.8E-1	g	market for sulfur - GLO
CMK-3	2.2E-1	n/a	2.2E-1	1.0E-1	2.2E-1	n/a	g	Section S3.5
Carbon black	n/a	2.2E-1	n/a	n/a	n/a	1.0E-1	g	market for carbon black - GLO
PVDF	1.3E-1	n/a	1.3E-1	1.0E-1	1.3E-1	n/a	g	market for polyvinylfluoride - GLO
PEG	n/a	1.3E-1	n/a	n/a	n/a	1.0E-1	g	Section S3.3
Acetonitrile	4.4E-2	4.4E-2	4.4E-2	6.3E-2	4.4E-2	6.3E-2	g	market for acetonitrile - GLO
Aluminum foil	3.8E-1	3.8E-1	3.8E-1	1.2E-1	3.8E-1	1.2E-1	g	Chordia et al. [27]
**Energy and process inputs**								
Electricity	6.3E-3	6.3E-3	6.3E-3	6.3E-3	6.3E-3	6.3E-3	kWh	Scenario dependent, see Table S39
District heating	4.9E-4	4.9E-4	4.9E-4	4.9E-4	4.9E-4	4.9E-4	MJ	market for heat, district or industrial, natural gas – RoW
District cooling	1.6E-2	1.6E-2	1.6E-2	1.6E-2	1.6E-2	1.6E-2	MJ	market for cooling energy - GLO
**Product output**								
Cathode	1.0E0	1.0E0	1.0E0	1.0E0	1.0E0	1.0E0	g	-
**Waste outputs**								
Organic waste	5.4E-2	5.4E-2	5.4E-2	6.3E-2	5.4E-2	6.3E-2	g	market for spent solvent mixture - RoW

S3.6.4 Electrolyte mixing and feeding

Electrolyte salts and solvents are mixed and then fed to the stage where Li-S cells are assembled. Energy and process inputs are obtained from Chordia et al. [27], but the material inputs are instead based on the material requirements of the studied Li-S cells. Some additives are included in the original dataset. Since we do not know if those are needed also for Li-S electrolytes, and since they are used in very low amounts, these are excluded in this modeling. The unit-process table for this process is presented in Table S20 and the flowchart in Figure S10.

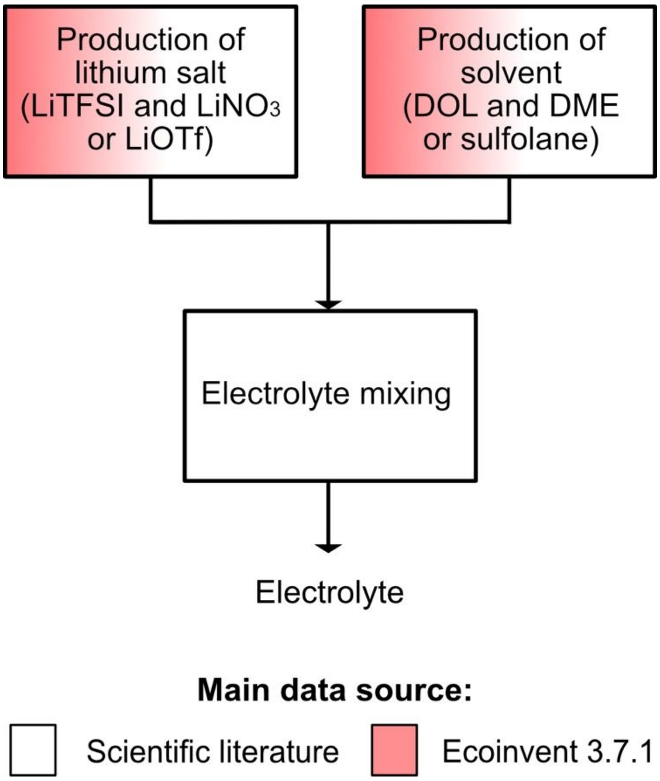

Figure S10. Flowchart of electrolyte production.

Table S20. Unit-process table for mixing and feeding the electrolyte. B=base scenario, M=material selection scenario, E=energy system scenario, T=technical performance scenario, R=recycling scenario, C=combined scenario, n/a= not applicable.

Material inputs	B	M	E	T	R	C	Unit	Upstream data source
LiTFSI	2.8E-1	n/a	2.8E-1	2.8E-1	2.8E-1	n/a	g	Section S3.1
LiOTf	n/a	1.5E-1	n/a	n/a	n/a	1.5E-1	g	Section S3.2
DOL	3.2E-1	n/a	3.2E-1	3.2E-1	3.2E-1	n/a	g	Deng et al. [1]
DME	3.9E-1	n/a	3.9E-1	3.9E-1	3.9E-1	n/a	g	ethylene glycol dimethyl ether production - RoW
Sulfolane	n/a	8.5E-1	n/a	n/a	n/a	8.5E-1	g	Section S3.4
Lithium nitrate	1.8E-2	n/a	1.8E-2	1.8E-2	1.8E-2	n/a	g	Deng et al. [1]
**Energy and process inputs**								
Electricity	2.5E-5	2.5E-5	2.5E-5	2.5E-5	2.5E-5	2.5E-5	kWh	Scenario dependent, see Table S39
District heating	4.2E-4	4.2E-4	4.2E-4	4.2E-4	4.2E-4	4.2E-4	MJ	market for heat, district or industrial, natural gas - RoW
District cooling	3.6E-4	3.6E-4	3.6E-4	3.6E-4	3.6E-4	3.6E-4	MJ	market for cooling energy - GLO
Nitrogen	1.6E0	1.6E0	1.6E0	1.6E0	1.6E0	1.6E0	g	market for nitrogen, liquid - RoW
**Product output**								
Electrolyte	1.0E0	1.0E0	1.0E0	1.0E0	1.0E0	1.0E0	g	-

S3.6.5 Cell assembly and formation

In Chordia et al. [27], the cell assembly is initiated by winding of the cathode and anode rolls. While that study models production of cylindrical cells, this study models pouch cell production and therefore there are some differences in the assembly process. For pouch cells, the slitted rolls are cut into individual electrodes, using e.g. shear cutting or laser cutting.[28] Electrodes are then stacked on top of each other, with a separator in between. Since the tabs can be welded in conjunction with either the slitting step or in the cell assembly according to Chordia et al. [27], the latter is assumed in this case. The stack is placed inside the cell container, in this case a pouch consisting of PE and aluminum, which is then sealed. The pouch is filled with electrolyte under vacuum, which is distributed throughout the cell using a wetting or soaking step. Chordia et al. [27] also model a washing step to remove spilled electrolyte. The most apparent difference in the assembly process for a pouch cell compared to a cylindrical cell appears to be the cutting and stacking process instead of winding the electrode rolls.

Jinasena et al. [28] also mention that the wetting process can be done differently, depending on the cell geometry. While there are differences, we assume that the energy requirement will be similar regardless of the difference in subprocesses (e.g., cutting and stacking versus winding). Therefore, we use the electricity requirement as reported in Chordia et al. [27], although the amounts of the different cell components are scaled to match the cell weight modeled in this study. Also, the amount of water required for the washing step is scaled to the weight of a Li-S cell. The unit-process table for this process is presented in Table S21 and the flowchart in Figure S11.

While Jinasena et al. [28] state that the formation procedure is dependent on cell design and chemistry, it is not known how an industrial forming process for Li-S cells differs from the one modeled in Chordia et al. [27] for LIBs. We therefore assume the same electricity requirement as in Chordia et al. [27]. A small number of cells get damaged during the formation, but since the share of Li-S cells damaged during the formation process is uncertain, we neglect this loss. The unit-process table for this process is presented in Table S22 and the flowchart in Figure S10.

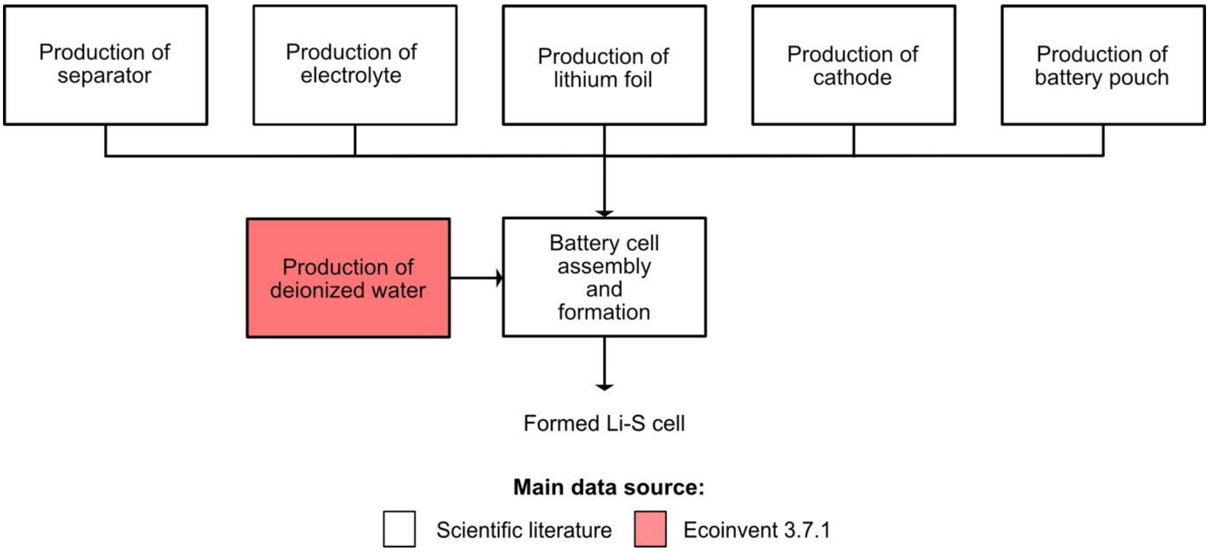

Figure S11. Flowchart of Li-S cell production.

Table S21. Unit-process table for cell assembly. B=base scenario, M=material selection scenario, E=energy system scenario, T=technical performance scenario, R=recycling scenario, C=combined scenario.

Material inputs	B	M	E	T	R	C	Unit	Upstream data source
Lithium foil	4.0E0	4.0E0	4.0E0	7.4E0	4.0E0	7.4E0	g	Section S3.6.1
Separator	6.0E0	6.0E0	6.0E0	4.6E0	6.0E0	4.6E0	g	Section S3.6.2
Cathode	8.0E0	8.0E0	8.0E0	1.1E1	8.0E0	1.1E1	g	Section S3.6.3
Electrolyte	2.9E1	2.9E1	2.9E1	2.0E1	2.9E1	2.0E1	g	Section S3.6.4
Battery pouch	1.3E-1	1.3E-1	1.3E-1	1.3E-1	1.3E-1	1.3E-1	g	Ellingsen et al. [33]
Negative tab	1.2E-1	1.2E-1	1.2E-1	1.2E-1	1.2E-1	1.2E-1	g	Ellingsen et al. [33]
Positive tab	6.9E-2	6.9E-2	6.9E-2	6.9E-2	6.9E-2	6.9E-2	g	Ellingsen et al. [33]
Water, deionized	3.1E1	3.1E1	3.1E1	3.1E1	3.1E1	3.1E1	g	market for water, deionised – RoW
**Energy and process inputs**								
Electricity	4.6E-2	4.6E-2	4.6E-2	4.6E-2	4.6E-2	4.6E-2	kWh	Scenario dependent, see Table S39
**Product output**								
Battery cell	1.0E0	1.0E0	1.0E0	1.0E0	1.0E0	1.0E0	item	-
**Waste outputs**								
Wastewater	3.1E1	3.1E1	3.1E1	3.1E1	3.1E1	3.1E1	g	market for wastewater, average | wastewater, average

Table S22. Unit-process table for the cell formation.

Material inputs	Normalized to unit process	Unit	Upstream data source
Li-S cell	1.00E0	item	Section 3.6.5
**Energy and process inputs**			
Electricity	2.25E-1	kWh	Scenario dependent, see Table S39
**Product outputs**			
Formed cell	1.00E0	item	-

S3.6.6 1 kWh storage capacity

1 kWh of theoretical storage capacity is the functional unit for the cradle-to-gate system. A cell weight of 46 g is modeled, due to the slight difference in cell weight of the different cell compositions (see Table 2 in the main manuscript). The number of formed cells required to provide the function depends on the specific energy density. Consequently, fewer cells are

required in the technical performance and combined scenarios because of the higher specific energy density considered in those scenarios. Also, the battery factory construction and operation are added in this process, where a combination of a precious metal refinery and an electronics factory has been used as proxy for the construction, following Chordia et al. [27]. The unit-process table for this process is presented in Table S23.

Table S23. Unit-process table for production of 1 kWh storage capacity. B=base scenario, M=material selection scenario, E=energy system scenario, T=technical performance scenario, R=recycling scenario, C=combined scenario.

Inputs	B	M	E	T	R	C	Unit	Upstream data source
Formed cell	1.45E2	1.45E2	1.45E2	4.35E1	1.45E2	4.35E1	item	See Section S3.6.5
Facility inputs								
Factory construction and operation	5.00E-7	5.00E-7	5.00E-7	5.00E-7	5.00E-7	5.00E-7	item	Chordia et al. [27]
Product output								
Installed capacity	1.00E0	1.00E0	1.00E0	1.00E0	1.00E0	1.00E0	kWh	-

S3.7 Battery module production

The size (41 litre) and total weight (25 kg) of a battery module, as well as the total battery cell weight in one module (19 kg), are obtained from Ainsworth [34]. The housing consists of an outer frame in steel and PP as insulation material. The electric components consist of busbars, whose function is to carry and distribute electricity. Furthermore, a printed circuit board (PCB) is required, but as it is part of the battery management system (BMS), it will be described in the next subsection. Whether the battery cells are connected in series or parallel is disregarded in this modeling due to the lack of information and since the total available energy (measured in Wh) will be the same regardless of how the cells are connected. All components are assembled into a module, which requires energy in the form of heat and electricity. The total cell weight for one module (which is about 75% of the total module weight) is used in the modeling, but the weight of other module components including module housing, electric components are not provided in Ainsworth [34]. Therefore, the weight distribution of the remaining components besides the PP (0.10 kg steel/kg BM, 0.024 kg bimetallic busbar/kg BM, 0.011 kg aluminum

busbar/kg BM and 0.0034 kg copper busbar/kg BM, where BM = battery module) are obtained from Peters and Weil [35]. As the cell weight ratio is 85% in Peters and Weil [35], the PP is assumed to fill up the extra space in this modeling, with the motivation that pouch cells might need more housing structure than the cylindrical cells modeled in Peters and Weil [35]. The electricity and heat values reported in Peters and Weil [35] consider both cell and module manufacturing, where cell production is reported to account for the majority of both. As the proportion of the module alone is not reported, no heat or electricity is included in the unit process. The unit-process table for this process is presented in Table S24 and the flowchart in Figure S12.

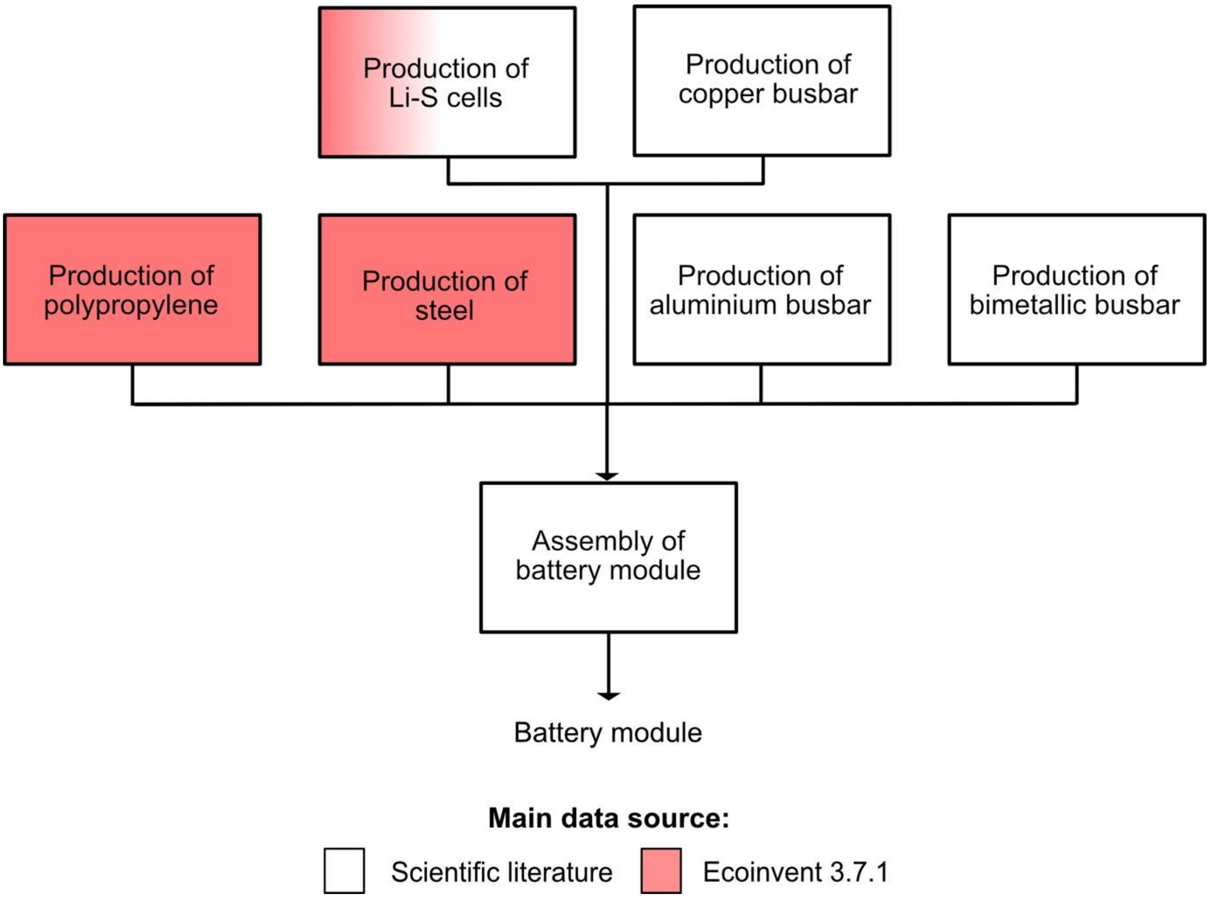

Figure S12. Flowchart of battery module production.

Table S24. Unit-process table for production of the battery module.

Inputs	Normalized to unit process	Unit	Upstream data source
Formed cells	4.08E2	item	Section S3.6.5
Aluminum busbar	2.75E-1	kg	Ellingsen et al. [33]
Bimetallic busbar	5.93E-1	kg	Ellingsen et al. [33]
Copper busbar	8.50E-2	kg	Ellingsen et al. [33]
Polypropylene	2.75E0	kg	market for polypropylene, granulate - GLO
Steel	2.50E0	kg	market for steel, low-alloyed - GLO
Facility and machinery inputs			
Injection moulding	2.75E0	kg	market for injection moulding - GLO
Metal working, for steel	2.50E0	kg	market for metal working, average for steel product manufacturing - GLO
Product output			
Battery module	1.00E0	item	-

S3.8 BMS production

The BMS consists of:

- rack-level PCBs (one for each rack), referred to as the integrated battery interface system (IBIS) in Ellingsen et al. [33],
- module-level PCBs (one for each module), referred to as battery module boards in Ellingsen et al. [33],
- fasteners,
- a high voltage system, and
- a low voltage system.

The composition as well as the assembly process of the PCBs are taken from Nordelöf and Alatalo [36], Nordelöf et al. [37] and Nordelöf [38], while the composition of the remaining components as well as the total BMS amount are obtained from Ellingsen et al. [33]. For the PCBs, a mass distribution modeled by Nordelöf and Alatalo [36] is used (see Table S25), which entails that the PCB input amounts reported in unit processes in Ellingsen et al. [33] are assumed to be the weight of an assembled PCB with all its components. Using the unmounted board weight, calculated using the mass distribution in Table S25, other circuit board components (surface mounted devices (SMD), solder and coating) can be scaled following the datasets in Nordelöf and Alatalo [36]. For rack-level PCBs, an external connector is assumed to be required in addition to the other components, for which assembly process is obtained from Nordelöf and

Alatalo [36]. Note that while the module-level PCBs are assembled during the battery module production, we here chose to gather all BMS related components in one unit process, following Ellingsen et al. [33]. The unit-process table for the BMS, expressed in BMS items per rack, is presented in Table S26, and the corresponding flowchart in Figure S13.

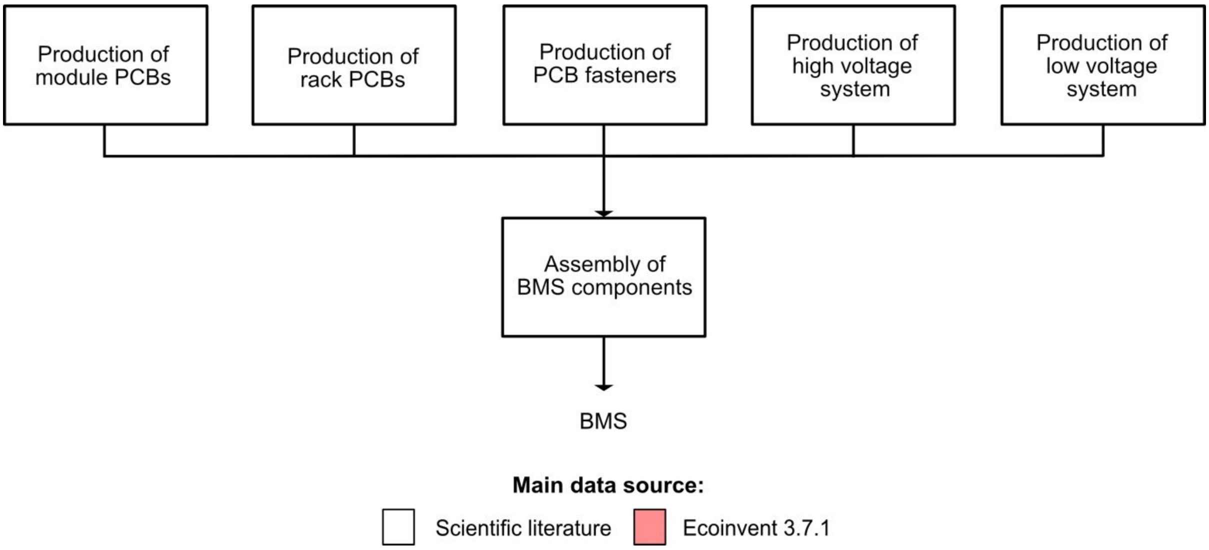

Figure S13. Flowchart of BMS production.

Table S25. Mass proportion of PCB components, obtained from Nordelöf and Alatalo [36]. SMD= surface mounted devices.

Circuit board component	Estimated mass proportion (wt%)
Unmounted board	44.7
SMD	53.7
Solder	1.00
Coating	0.600

Table S26. Unit-process table for production of the BMS (BMS for one rack).

Inputs	Normalized to unit process	Unit	Upstream data source
Module PCB	1.1E0	kg	Nordelöf and Alatalo [36]
Rack PCB	4.9E-1	kg	Nordelöf and Alatalo [36]
Rack PCB fasteners	2.8E-3	kg	Ellingsen et al. [33]
High voltage system	2.8E0	kg	Ellingsen et al. [33]
Low voltage system	1.2E0	kg	Ellingsen et al. [33]
Product output			
BMS	1.00E0	item	-

S3.9 Battery rack production

The input materials required to produce the battery rack are obtained from Peters and Weil [35]. However, the input amounts of each respective material, provided in per 1 kg of battery rack in that study, are not used for this unit process, except for the cable and factory amounts. Instead, more specific sources are used for obtaining information about exact input amounts of materials. In one battery rack, it is assumed that 16 modules fit, according to the configuration shown in Ainsworth [34]. The amount of BMS is based on Ellingsen et al. [33], see details in Section S3.8. The amount of cooling system, 0.11 kg/kWh installed capacity, is obtained from Pellow et al. [39]. The amount of rack housing is approximated based on outer dimensions of the rack and the density of steel, and data on the production process of the rack housing is obtained from Peters and Weil [35]. Also the amount of facility inputs is based on the same study. Lastly, the amount of cable is estimated from the value per 1 kg of battery rack (0.0040 kg cable) from Peters and Weil [35] and scaled up to the weight of one battery rack. The unit-process table for this process is presented in Table S27 and the flowchart in Figure S14.

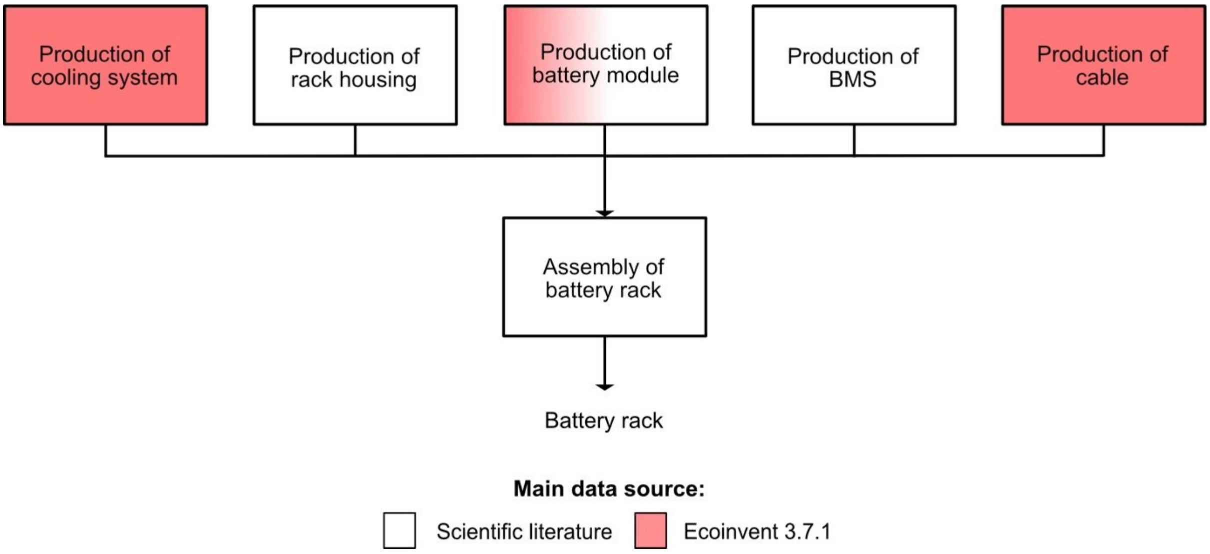

Figure S14. Flowchart of battery rack production.

Table S27. Unit-process table for production of the battery rack. B=base scenario, M=material selection scenario, E=energy system scenario, T=technical performance scenario, R=recycling scenario, C=combined scenario.

Material inputs	B	M	E	T	R	C	Unit	Upstream data source
Battery module	1.6E1	1.6E1	1.6E1	1.6E1	1.6E1	1.6E1	item	Section S3.7
BMS	1.0E0	1.0E0	1.0E0	1.0E0	1.0E0	1.0E0	item	Section S3.8
Cable	1.8E0	1.8E0	1.8E0	1.8E0	1.8E0	1.8E0	kg	market for cable, unspecified – GLO
Rack housing	5.0E1	5.0E1	5.0E1	5.0E1	5.0E1	5.0E1	kg	Peters and Weil [35]
Cooling system	5.0E0	5.0E0	5.0E0	1.7E1	5.0E0	1.7E1	kg	market for fan, for power supply unit, desktop computer – GLO
Facility inputs								
Metal working factory	2.1E-7	2.1E-7	2.1E-7	2.1E-7	2.1E-7	2.1E-7	item	market for metal working factory – GLO
Product output								
Battery rack	1.0E0	1.0E0	1.0E0	1.0E0	1.0E0	1.0E0	item	-

S3.10 Fire suppression system production

Pellow et al. [39] report that a fire suppression system is necessary for large stationary storage installations, due to the importance of mitigating potential thermal runaways. In Table S28, a simple unit process that only contains material inputs reported by Pellow et al. [39] is shown. It is assumed that carbon dioxide can function as fire suppressant, since it is used in fire extinguishers for putting out electrical fires.

Table S28. Unit-process table for production of the fire suppression system.

Material inputs	Normalized to unit process	Unit	Upstream data source
Steel	7.8E-1	kg	market for steel, low-alloyed – GLO
Fire suppressant	2.2E-1	kg	market for carbon dioxide, liquid – RoW
Product output			
Fire suppressant system	1.0E0	kg	-

S3.11 Installation production

The energy storage installation consists of a 40-foot intermodal shipping container standing on a concrete foundation, which contains battery racks, a fire suppression system and inverters. The number of battery racks that fit in the container is calculated based on the estimated outer dimensions of a battery rack (490 x 660 x 2200 mm). Since the lifetime of the installation is 20 years and 300 cycles per year is assumed, battery modules in the racks are assumed to be changed when the cells have reached their maximum cycle life, and the number of changes depend on the scenario. The remaining components are assumed to not be changed during the installation's lifetime. The amount of fire suppression system required is estimated as the proxy value given in Pellow et al. [39], which is 0.08 kg/kWh. Note that the amount of fire suppression system required increases with the capacity of the installation, i.e. the kWh output. The number and size of inverters are also dependent on the capacity, specifically the power capacity.

Energy storage installations used for integration of renewable energy sources need to have a discharge duration of several hours.[40] A C-rate of 0.1 is therefore selected, which equals a discharge duration of 10 hours, which together with storage capacities of 1800 or 6000 kWh (depending on the scenario), the power capacity equals to 180 or 600 kW. It might be that the larger amounts of components, such as the fire suppressant system and inverters for the technical performance and the combined scenario, means that the assumed 40-foot intermodal container cannot fit everything. However, since the exact physical dimensions of these non-battery components are unknown and due to the container's negligible contribution to all impact categories, this aspect is neglected. The installation is assumed to be manually assembled onsite. The unit-process table for this process is presented in Table S29 and the flowchart in Figure S15. A table showing the dimensioning and operational parameters for the installation is furthermore provided (Table S30).

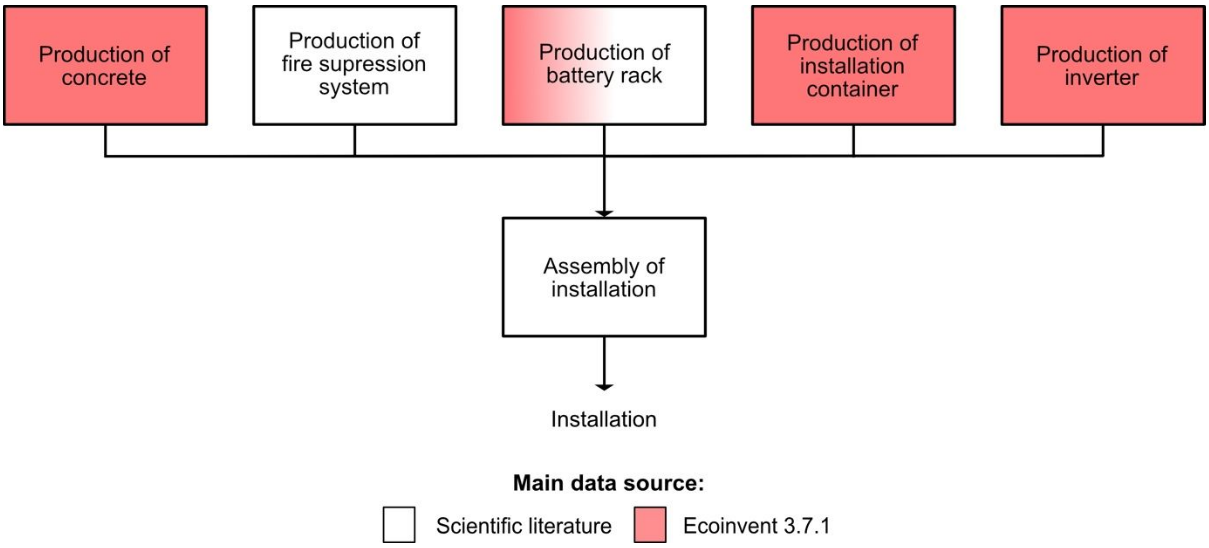

Figure S15. Flowchart of installation assembly.

Table S29. Unit-process table for assembly of the installation. B=base scenario, M=material selection scenario, E=energy system scenario, T=technical performance scenario, R=recycling scenario, C=combined scenario.

Material inputs	B	M	E	T	R	C	Unit	Upstream data source
Battery rack	4.00E1	4.00E1	4.00E1	4.00E1	4.00E1	4.00E1	item	Section S3.9
Battery module	1.92E3	1.92E3	1.92E3	6.40E2	1.92E3	6.40E2	item	Section S3.7
Concrete	1.50E1	1.50E1	1.50E1	1.50E1	1.50E1	1.50E1	m^3	market for concrete, normal – RoW
Fire suppression	1.40E2	1.40E2	1.40E2	4.80E2	1.40E2	4.80E2	kg	Section S3.10
Container	1.00E0	1.00E0	1.00E0	1.00E0	1.00E0	1.00E0	item	market for intermodal shipping container, 40-foot – GLO
Inverter	3.60E-1	3.60E-1	3.60E-1	1.20E0	3.60E-1	1.20E0	item	market for inverter, 500kW – GLO
**Product output**								
Installation	1.00E0	1.00E0	1.00E0	1.00E0	1.00E0	1.00E0	item	-

Table S30. Dimensioning and operational parameters for the installation. B=base scenario, M=material selection scenario, E=energy system scenario, T=technical performance scenario, R=recycling scenario, C=combined scenario.

Parameter	B	M	E	T	R	C	Unit
Installed capacity	1.8E3	1.8E3	1.8E3	6.0E3	1.8E3	6.0E3	kWh
Power	1.8E2	1.8E2	1.8E2	6.0E2	1.8E2	6.0E2	kW
Cycles per year	3.0E2	3.0E2	3.0E2	3.0E2	3.0E2	3.0E2	number
Installation lifetime	2.0E1	2.0E1	2.0E1	2.0E1	2.0E1	2.0E1	year
Number of module changes	4.0E0	4.0E0	4.0E0	2.0E0	4.0E0	2.0E0	number
Total delivered electricity*	5.8E3	5.8E3	5.8E3	2.5E4	5.8E3	2.5E4	MWh

*After 20 years of operation.

S3.12 Use phase

In the use phase, the installation is storing wind-based electricity, which is then delivered to the electric grid. The installed storage capacities (1800 kWh in the base, material selection, energy system and recycling scenarios and 6000 kWh in the technical performance and combined scenarios) need to be adjusted for losses occurring during operation. The roundtrip-efficiency as well as the inverter efficiency are included in this modeling, where the roundtrip-efficiency depends on the specific scenario (see Table 1 in the main manuscript) and an inverter efficiency of 95.4% is assumed, obtained from ecoinvent. Furthermore, a depth of discharge (DOD) of 80% is assumed,[41] which is the fraction of a battery's capacity, from a fully charged cell, that is removed during discharge.[42] The cycle life is negatively affected by deep cycles, and we therefore set a maximum DOD (which is assumed for all cycles), based on Battke et al. [41]. da Silva Lima et al. [43] report that a stationary storage installation consumes electricity, even in a stand-by mode. Therefore, the electricity requirement for operation is approximated as 100 kWh of electricity per MWh of storage capacity, following that study. Note that the *stored* electricity is solely wind-based since this is the function of the installation, i.e. to store and deliver wind power. However, the electricity *needed to operate* the installation can originate from different sources, according to the differente scenarios of this study (Table S39). As the project lifetime is 20 years, the values are adjusted to this time horizon. Furthermore, since it is not always windy, it is assumed that the batteries will be cycled 300 times per year, which equals approximately 300 days given the selected C-rate. The unit-process table for this process is presented in Table S31 and the flowchart in Figure S16.

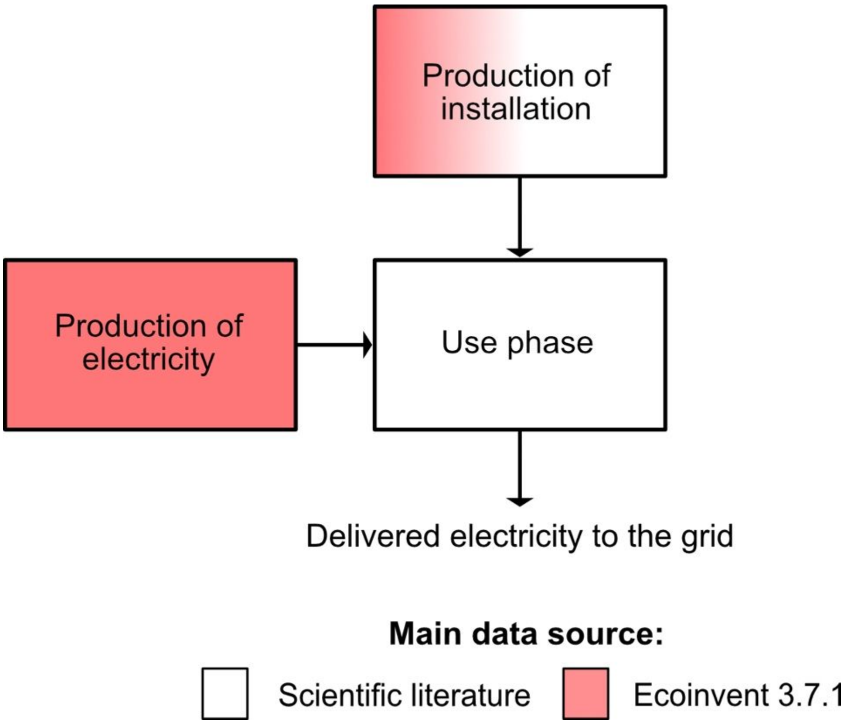

Figure S16. Flowchart of the use phase.

Table S31. Unit-process table for the use phase. B=base scenario, M=material selection scenario, E=energy system scenario, T=technical performance scenario, R=recycling scenario, C=combined scenario.

Inputs	B	M	E	T	R	C	Unit	Upstream data source
Installation	1.7E-4	1.7E-4	1.7E-4	4.0E-5	1.7E-4	4.0E-5	item	Section S3.11
Electricity, losses	8.7E-1	8.7E-1	8.7E-1	4.6E-1	8.7E-1	4.6E-1	MWh	electricity production, wind, 1-3MW turbine, onshore | electricity, high voltage*
Electricity, for operation	2.3E-1	2.3E-1	2.3E-1	1.8E-1	2.3E-1	1.8E-1	MWh	Scenario dependent, see Table S39
Product output								
Delivered electricity to the grid	1.0E0	1.0E0	1.0E0	1.0E0	1.0E0	1.0E0	MWh	-

*A voltage transformation process is added to change high voltage into medium voltage electricity.

S3.13 Disassembly and end-of-life treatment

Battery modules will be replaced when the cells have reached their maximum cycle life, which will occur four times for the base scenario, material selection scenario, energy system scenario and recycling scenario but only two times for the two other scenarios. In addition, the entire installation is assumed to be disassembled after 20 years, for which the unit-process table is shown in Table S32. Note that the total amount of battery modules used during the years is shown in the table. The electronic scrap consists of inverters and the BMS. Regarding the battery module replacement, the modules containing cells that have reached their maximum cycle life are assumed to be manually removed from the battery racks and transported to a recycling facility. The actual transportation is not included in the model. At the recycling facility, battery modules are assumed to be manually disassembled, which yields battery cells as well as other materials and elements, see Table S33. The battery cells are assumed to undergo a deactivation process. All disassembled non-cell components and materials, as well as some parts of the cells (see Figure S17), are assumed to go through a shredding and sorting process. All materials that cross the system boundary are modeled using the cutoff approach. The parts of the cell which remain after the cell separation are assumed to undergo a grinding process followed by further end-of-life (EoL) treatment. There are two different EoL processes modeled in this study, namely landfilling and hydrometallurgical treatment which are described in the following subsections. A flowchart showing the EoL system of this study is presented in Figure S17.

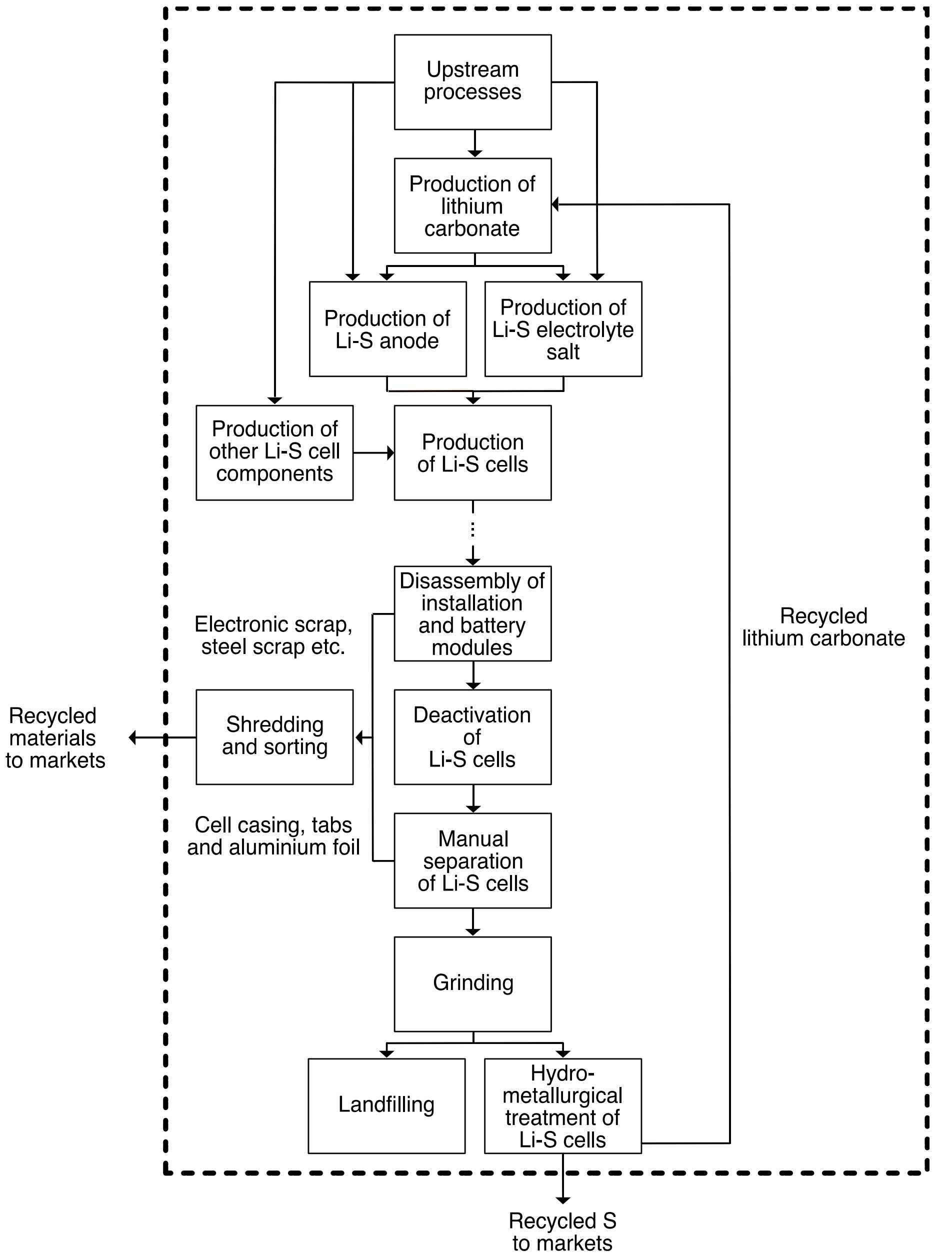

Figure S17. Flow chart of the EoL system considered in this study. S=sulfur.

Table S32. Unit-process table for disassembly of installation. B=base scenario, M=material selection scenario, E=energy system scenario, T=technical performance scenario, R=recycling scenario, C=combined scenario.

Material inputs	B	M	E	T	R	C	Unit	Upstream data source
Installation	1.0E0	1.0E0	1.0E0	1.0E0	1.0E0	1.0E0	item	Section S3.11
Recyclable outputs								
Battery modules	2.6E3	2.6E3	2.6E3	1.3E3	2.6E3	1.3E3	item	-
Steel scrap*,**	5.8E3	5.8E3	5.8E3	6.1E3	5.8E3	6.1E3	kg	-
Electronic scrap*,**	1.0E3	1.0E3	1.0E3	2.9E3	1.0E3	2.9E3	kg	-

*Modeled as cutoff. **Scrap coming from the battery modules is not included here.

Table S33. Unit-process table for disassembly of a battery module.

Material inputs	Normalized to unit process	Unit	Upstream data source
Battery module	1.0E0	item	Section S3.7
Recyclable outputs			
Battery cell	4.1E2	item	-
Aluminum scrap*	4.0E-1	kg	-
Copper scrap*	4.1E-1	kg	-
Steel scrap*	2.5E0	kg	-
Electronic scrap*	6.9E-2	kg	-
Waste outputs			
Plastic waste	2.8E0	kg	market for waste plastic, consumer electronics - RoW

*Modeled as cutoff.

S3.13.1 Shredding and sorting

Disassembled components and materials coming from the container, racks, modules, BMS, electronics and some parts of the cells are modeled to be shredded using a hammer mill.[44] The shredded materials are then separated using methods such as air separation and magnetic separation, which are followed by further treatment processes that yield recyclable material scrap. The unit-process table for shredding and sorting is shown in Table S34. Note that all components and materials undergoing shredding and sorting have been collapsed into one mass flow. The composition of the flows collapsed can be found in Tables S32, S33 and S36.

Table S34. Unit-process table for shredding and sorting.

Material inputs	Normalized to unit process	Unit	Upstream data source
Disassembled components	1.0E0	kg	Section S3.13
Energy and process inputs			
Electricity	5.4E-2	kWh	Scenario dependent, see Table S39
District heating	3.2E-2	MJ	market for heat, district or industrial, natural gas - RoW
Recyclable outputs			
Recyclable material scrap	1.0E0	kg	-

S3.13.2 Cell deactivation

As there are several flammable materials in Li-S cells, a deactivation step is modeled to ensure a safe handling without uncontrolled thermal runaway.[45, 46] The deactivation is assumed to occur through pyrolysis.[45] During pyrolysis, 27.6 wt% is lost on average, mainly from the carbon-based electrolyte, binder and separator. The carbon-based off-gases from the pyrolysis are assumed to be emitted to air as non-methane volatile organic compounds (NMVOC). The cell weight after pyrolysis is calculated by subtracting the average loss from the cell weight. The unit-process table for cell deactivation is shown in Table S35.

Table S35. Unit-process table for cell deactivation. B=base scenario, M=material selection scenario, E=energy system scenario, T=technical performance scenario, R=recycling scenario, C=combined scenario.

Material inputs	B	M	E	T	R	C	Unit	Upstream data source
Battery cell	1.0E0	1.0E0	1.0E0	1.0E0	1.0E0	1.0E0	kg	Section S3.13
Energy inputs								
Electricity	2.6E-1	2.6E-1	2.6E-1	2.6E-1	2.6E-1	2.6E-1	kWh	Scenario dependent, see Table S39
Product output								
Deactivated cells	7.2E-1	7.2E-1	7.2E-1	7.2E-1	7.2E-1	7.2E-1	kg	-
Process emissions								
NMVOC	2.8E-1	2.8E-1	2.8E-1	2.8E-1	2.8E-1	2.8E-1	kg	-

S3.13.3 Cell separation and grinding

Following the pyrolysis, there is a separation process and then a grinding process. During the separation process, the casing (cell pouch), the aluminum foil and the tabs are removed,[45] which are modeled to go through the same shredding and sorting process as the non-cell components. Both the separation and grinding are reported to be performed in an inert environment, using a glove box to prevent the lithium from oxidizing. Despite this measure, the lithium foil cannot be recovered intact from the Li-S cell, since the lithium has been transformed into lithium sulfide in a discharged cell. In addition, any remaining lithium metal will decompose to other lithium compounds during pyrolysis. Regarding the separation process, we assume that 10% of the original aluminum content is left, since there is not enough information regarding how much aluminum remains after this process. In terms of the inert atmosphere required, we assume that a dry room can be used, since lithium anode production can occur both in dry rooms and inert environments according to Schnell et al. [30]. The energy requirement of the dry room is again modeled based on personal communication with the first author of Chordia et al. [27]. The unit-process table for cell separation and grinding is shown in Table S36.

Table S36. Unit-process table for cell separation and grinding. B=base scenario, M=material selection scenario, E=energy system scenario, T=technical performance scenario, R=recycling scenario, C=combined scenario.

Material inputs	B	M	E	T	R	C	Unit	Upstream data source
Deactivated cells	1.0E0	1.0E0	1.0E0	1.0E0	1.0E0	1.0E0	kg	Section S3.13.2
Energy and process inputs								
Electricity	1.6E0	1.6E0	1.6E0	1.6E0	1.6E0	1.6E0	kWh	Scenario dependent, see Table S39
District heating	5.0E0	5.0E0	5.0E0	5.0E0	5.0E0	5.0E0	MJ	market for heat, district or industrial, natural gas - RoW
District cooling	4.2E0	4.2E0	4.2E0	4.2E0	4.2E0	4.2E0	MJ	market for cooling energy - GLO
Product output								
Grinded cells	9.1E-1	9.1E-1	9.1E-1	9.5E-1	9.1E-1	9.5E-1	kg	-
Recyclable outputs								
Aluminum scrap*	8.6E-2	8.6E-2	8.6E-2	4.4E-2	8.6E-2	4.4E-2	kg	-
Copper scrap*	3.6E-3	3.6E-3	3.6E-3	3.6E-3	3.6E-3	3.6E-3	kg	-

*Modeled as cutoff.

S3.13.4 Landfilling

After the cells (remains after the separation) have been grinded, they are modeled to be sent to a landfill in the base, material selection, energy system and technical performance scenarios. The landfill is assumed to be an inert material landfill, for which the data is obtained from Ecoinvent. The unit-process table for landfilling is shown in Table S37.

Table S37. Unit-process table for landfilling. B=base scenario, M=material selection scenario, E=energy system scenario, T=technical performance scenario.

Material inputs	B	M	E	T	Unit	Upstream data source
Grinded cells	1.0E0	1.0E0	1.0E0	1.0E0	kg	Section S3.13.3
Process-specific burdens, inert material landfill	1.0E0	1.0E0	1.0E0	1.0E0	kg	market for process-specific burdens, inert material landfill - RoW
Facility inputs						
Inert material landfill construction	1.5E-9	1.5E-9	1.5E-9	1.5E-9	item	market for inert material landfill - GLO

S3.13.5 Hydrometallurgical treatment

Recycling through hydrometallurgy is used to extract valuable metals, in this case lithium, from the Li-S cells. The specific process is modeled based on Schwich et al. [45]. The entire process (from grinded cells to extracted lithium) is shown in Figure S18, and the unit-process in Table S38.

After a sieving step, which yields a residue, the mass is subjected to leaching, where sodium hydroxide is selected as the leaching agent due to high lithium yield and a low number of impurities.[45] Hydrogen peroxide is also added during the leaching. The leaching temperature and time are 60°C and 1 hour, respectively. Furthermore, a solid/liquid ratio of 1/50 is applied, where solid is the amount of residue and liquid is the leaching agent. Sulfuric off-gases, such as hydrogen sulfide and sulfur dioxide, are released during leaching. After the leaching, the leachate is filtrated, from which a carbon-rich filter cake is formed. The next step is to remove the remaining amount of aluminum, which is done by adjusting the pH with nitric acid. Hydrogen peroxide is again added. Aluminum is then precipitated as aluminum hydroxide, which is removed by filtration. Sodium carbonate is added to the filtrate, functioning as the carbonate source for producing lithium carbonate, see SR16. There is also a second pH adjustment, where nitric acid is again used. The temperature is increased to 100°C, causing

precipitation of lithium carbonate, which is then isolated by filtration. A lithium yield of 85% for the hydrometallurgical part of the process is reported.

$$2\ Li^{+} + Na_2CO_3 \longrightarrow Li_2CO_3 + 2\ Na^{+} \qquad (SR16)$$

Schwich et al. [45] report that the lithium carbonate contains 14.6 wt% impurities in total. However, the lithium carbonate used for lithium production require a lower share of impurities (<0.5%). Therefore, a purification step is added, which is modeled based on the patent by Perez et al. [19]. The purification is done by first dissolving the lithium carbonate cake in cold water and then the solution is stirred. Carbon dioxide is injected, which reacts with water and forms carbonic acid, see SR17. Lithium carbonate then reacts with carbonic acid to form lithium bicarbonate (SR18):

$$H_2O + CO_2 \longrightarrow H_2CO_3 \qquad (SR17)$$

$$H_2CO_3 + Li_2CO_3 \longrightarrow 2LiHCO_3 \qquad (SR18)$$

Lithium bicarbonate is more soluble in water than is lithium carbonate, which is why the conversion to the bicarbonate is performed. The reaction mixture is filtered to remove impurities. The filtrate is then heated, which makes lithium bicarbonate decompose into lithium carbonate, see SR19. Another filtration, washing the lithium carbonate filter cake with water and drying yields battery-grade (>99.5%) lithium carbonate. The carbon dioxide produced in SR19 can be recycled,[19] and is thus assumed to be so.

$$2\ LiHCO_3 + heat \longrightarrow Li_2CO_3 + H_2O + CO_2 \qquad (SR19)$$

In terms of the modeled unit process, all energy requirements for the entire recycling process are calculated according to Piccinno et al. [9]. The input of sodium hydroxide added in the leaching is obtained from the solid/liquid ratio, and the hydrogen peroxide input is reported by Schwich et al. [45]. Regarding the sulfuric off-gases, they will likely be recovered as e.g. sulfuric acid in the future (rather than emitted to air). They are therefore considered a recyclable material and modeled as a sulfur product flow leaving the system. Since the cutoff approach to recyclable materials are applied for all materials except lithium in this study, the recovery of

the sulfur is not further modeled. We assume that all sulfur leaves the system in this step. In the first filtration, the remaining carbon is assumed to be removed and sent to solid waste treatment. After the filtration comes a pH adjustment, for which nitric acid and its concentration are reported, but the amount added is not. Therefore, we approximate this input amount by assuming the same amount as that reported for hydrogen peroxide. All remaining aluminum is assumed to precipitate as aluminum hydroxide after the pH adjustment, and removed by filtration. As can be seen in Figure S18, aluminum hydroxide nuclei are added to the first pH adjustment step and since there is no information considering the added amount, we assume that effectively all precipitated aluminum hydroxide is recycled as nuclei. Consequently, no aluminum hydroxide waste is assumed to be generated from the second filtration. In terms of the second pH adjustment, the same amount of nitric acid as in the previous pH adjustment step is assumed, due to lack of information. Regarding the carbonation, the amount of sodium carbonate added is calculated stoichiometrically based on the amount of lithium present in the reaction mixture. Since there are no indications of lithium losses during the pyrolysis, grinding or separation, we assume these losses to be negligible. Regarding the thermal precipitation, the temperature is provided in Schwich et al. [45], but the reaction time is not. Thus, one hour is assumed. In the purification step, a stochiometric amount of carbon dioxide input is calculated based on the amount of lithium carbonate obtained from the previous step, adjusting for a reaction yield of 95%.[14] However, Perez et al. [19] report that carbon dioxide can be recycled but not how efficiently, and hence we assume a recycling rate of 68% as for solvents according to Piccinno et al. [9]. The amount of water required for dissolving lithium carbonate is approximated based on the bicarbonate's solubility in water at 15°C. For SR18, a reaction yield of 95% is assumed.[14] In the washing stage, the water requirement is approximated by assuming 10 ml per g of lithium carbonate and washing step.

The recycling process is assumed to be independent of the cell composition, since it is likely that a future Li-S recycling facility will receive cells with various cell compositions. However, the higher lithium content in the combined scenario is considered since that metal is the focus of this study. Therefore, the sodium carbonate input varies in the different scenarios since a stochiometric amount is calculated, as well as the amount of carbon dioxide and water due to the different amounts of lithium carbonate produced (as per SR16, SR17, SR18 and SR19).

Table S38. Unit-process table for the hydrometallurgical treatment of grinded Li-S cells. R=recycling scenario, C=combined scenario.

Material inputs	R	C	Unit	Upstream data source
Grinded cell	1.0E0	1.0E0	kg	Section S3.13.3
Sodium hydroxide	6.4E0	6.4E0	kg	market for sodium hydroxide, without water, in 50% solution state - GLO
Sodium carbonate	1.1E0	1.8E0	kg	market for soda ash, dense - GLO
Nitric acid	3.3E-1	3.3E-1	kg	market for nitric acid, without water, in 50% solution state - RoW
Hydrogen peroxide	1.9E-1	1.9E-1	kg	market for hydrogen peroxide, without water, in 50% solution state - RoW
Carbon dioxide	1.2E-1	2.0E-1	kg	market for carbon dioxide, in chemical industry - GLO
Water, deionized	6.4E1	8.1E1	kg	market for water, deionised - RoW
**Energy and process inputs**				
Electricity	8.3E-3	8.3E-3	kWh	Scenario dependent, see Table S39
Steam	1.7E1	1.7E1	kg	market for steam, in chemical industry - RoW
**Product output**				
Lithium carbonate	6.1E-1	1.0E0	kg	-
**Recyclable outputs**				
Sulfur	7.4E-2	2.7E-1	kg	-
**Process emissions**				
Carbon dioxide	1.2E-1	2.0E-1	kg	-
**Waste outputs**				
Wastewater	7.2E1	8.9E1	kg	market for wastewater, average - RoW
Solid waste	4.0E-1	4.0E-1	kg	market for municipal solid waste, RoW

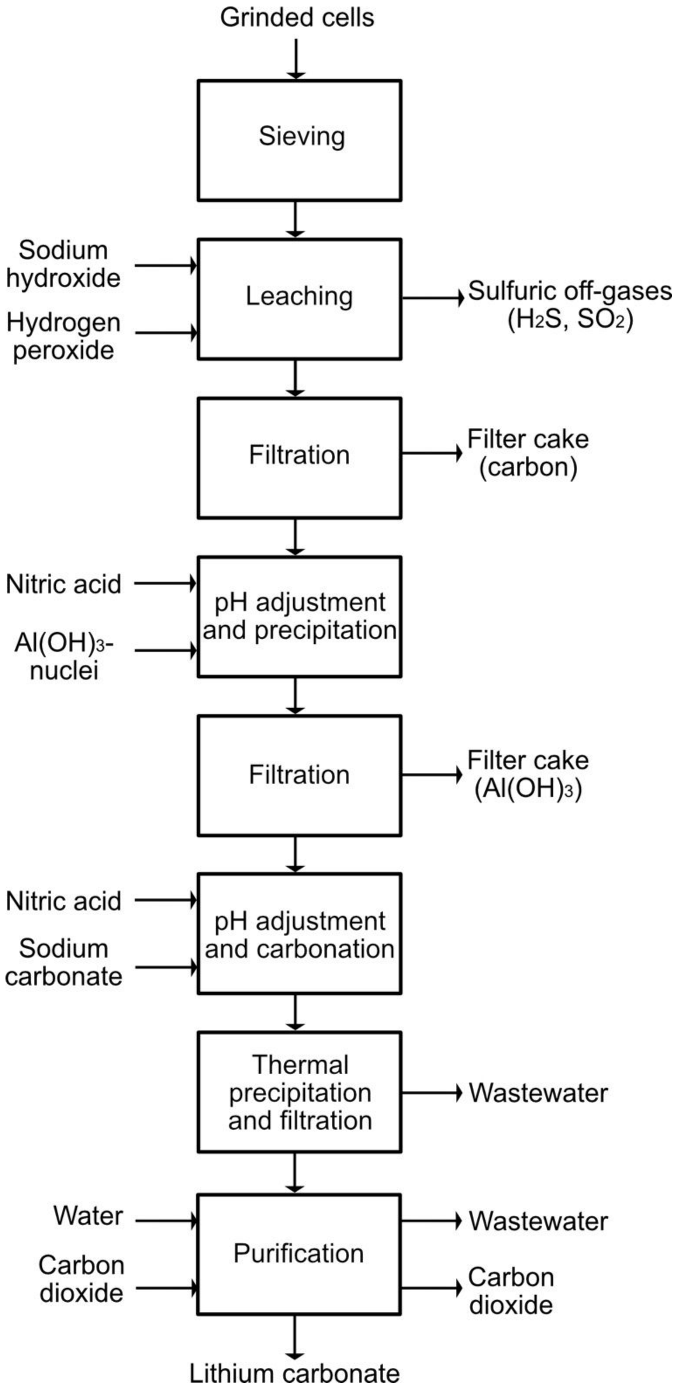

Figure S18. Process flowchart of hydrometallurgical treatment of Li-S cells.

S3.14 Electricity supply

In Table S39, upstream data sources regarding the electricity supply for different scenarios are provided.

Table S39. Electricity supply for the different scenarios.

Scenario	Upstream data source
Base scenario	market group for electricity, medium voltage | electricity, medium voltage – Europe without Switzerland
Material selection scenario	market group for electricity, medium voltage | electricity, medium voltage – Europe without Switzerland
Energy system scenario	electricity production, wind, 1-3MW turbine, onshore | electricity, high voltage*
Technical performance scenario	market group for electricity, medium voltage | electricity, medium voltage – Europe without Switzerland
Combined scenario	electricity production, wind, 1-3MW turbine, onshore | electricity, high voltage*

*A voltage transformation process is added to change high voltage into medium voltage electricity.

S4 LCIA results

In Tables S40 and S41, impact assessment results are provided for climate change, water consumption, mineral resource scarcity (crustal scarcity indicator and surplus ore potential), terrestrial acidification, freshwater eutrophication, ozone formation (for which human health and terrestrial ecosystem results are aggregated), fine particulate matter formation, stratospheric ozone depletion and fossil resource scarcity.

Table S40. LCIA results on cradle-to-gate level for climate change (CC), water consumption (WC), crustal scarcity indicator (CSI), surplus ore potential (SOP), terrestrial acidification (TA), freshwater eutrophication (FE), ozone formation (OF), fine particulate matter formation (PF), stratospheric ozone depletion (SOD) and fossil resource scarcity (FRS). B=base scenario, M=material selection scenario, E=energy system scenario, T=technical performance scenario, C=combined scenario.

Impact category	B	M	E	T	C	Unit
CC	3.04E2	1.12E2	2.51E2	7.94E1	2.92E1	kg CO_2 eq
WC	3.93E0	1.51E0	3.02E0	1.09E0	4.22E-1	m^3
CSI	6.31E4	3.97E4	5.74E4	2.36E4	1.74E4	kg Si eq
SOP	5.81E0	5.53E0	5.79E0	2.97E0	2.91E0	kg Cu eq
TA	1.05E0	5.42E-1	8.50E-1	2.81E-1	1.38E-1	kg SO_2 eq
FE	1.21E-1	6.44E-2	6.67E-2	3.49E-2	1.37E-2	kg P eq
OF	1.28E0	4.64E-1	1.10E0	3.45E-1	1.38E-1	kg NOx eq
PF	4.43E-1	2.20E-1	3.64E-1	1.22E-1	6.11E-2	kg PM2.5 eq
SOD	9.24E-3	4.24E-5	9.21E-3	1.99E-3	1.13E-5	kg CFC11 eq
FRS	8.01E1	3.37E1	6.57E1	2.09E1	8.48E0	kg oil eq

Table S41. LCIA results on cradle-to-grave level for climate change (CC), water consumption (WC), crustal scarcity indicator (CSI), surplus ore potential (SOP), terrestrial acidification (TA), freshwater eutrophication (FE), ozone formation (OF), fine particulate matter formation (PF), stratospheric ozone depletion (SOD) and fossil resource scarcity (FRS). B=base scenario, M=material selection scenario, E=energy system scenario, T=technical performance scenario, R=recycling scenario, C=combined scenario.

Impact category	B	M	E	T	R	C	Unit
CC	5.27E2	2.87E2	3.67E2	1.27E2	6.10E2	4.23E1	kg CO_2 eq
WC	7.03E0	4.03E0	4.30E0	1.92E0	8.36E0	5.61E-1	m^3
CSI	1.15E5	8.54E4	9.75E4	2.70E4	1.24E5	1.62E4	kg Si eq
SOP	7.79E0	7.44E0	7.73E0	1.62E0	3.07E0	6.08E-1	kg Cu eq
TA	1.84E0	1.21E0	1.24E0	4.61E-1	2.08E0	1.56E-1	kg SO_2 eq
FE	2.82E-1	2.12E-1	1.19E-1	9.98E-2	3.05E-1	2.04E-2	kg P eq
OF	3.30E0	2.28E0	2.73E0	6.47E-1	3.61E0	3.30E-1	kg NOx eq
PF	7.84E-1	5.06E-1	5.46E-1	1.97E-1	9.13E-1	7.74E-2	kg PM2.5 eq
SOD	1.16E-2	1.20E-4	1.15E-2	1.01E-3	1.17E-2	2.83E-5	kg CFC11 eq
FRS	1.40E2	8.21E1	9.64E1	3.39E1	1.61E2	1.13E1	kg oil eq

References

1. Deng, Y.; Li, J.; Li, T.; Gao, X.; Yuan, C., Life cycle assessment of lithium sulfur battery for electric vehicles. Journal of Power Sources 2017, 343, 284-295, DOI 10.1016/j.jpowsour.2017.01.036.
2. Arvidsson, R.; Janssen, M.; Svanström, M.; Johansson, P.; Sandén, B. A., Energy use and climate change improvements of Li/S batteries based on life cycle assessment. Journal of Power Sources 2018, 383, 87-92, DOI 10.1016/j.jpowsour.2018.02.054.
3. Cerdas, F.; Titscher, P.; Bognar, N.; Schmuch, R.; Winter, M.; Kwade, A.; Herrmann, C., Exploring the Effect of Increased Energy Density on the Environmental Impacts of Traction Batteries: A Comparison of Energy Optimized Lithium-Ion and Lithium-Sulfur Batteries for Mobility Applications. Energies 2018, 11 (1, 150), 1-20, DOI 10.3390/en11010150.
4. Wolff, D.; Canals Casals, L.; Benveniste, G.; Corchero, C.; Trilla, L., The Effects of Lithium Sulfur Battery Ageing on Second-Life Possibilities and Environmental Life Cycle Assessment Studies. Energies 2019, 12 (2440), 1-19, DOI 10.3390/en12122440.
5. Lopez, S.; Akizu-Gardoki, O.; Lizundia, E., Comparative life cycle assessment of high performance lithium-sulfur battery cathodes. Journal of Cleaner Production 2021, 282 (124528), 1-11, DOI 10.1016/j.jclepro.2020.124528.
6. Benveniste, G.; Sánchez, A.; Rallo, H.; Corchero, C.; Amante, B., Comparative life cycle assessment of Li-Sulphur and Li-ion batteries for electric vehicles. Resources, Conservation and Recycling Advances 2022, 15 (200086), 1-12, DOI 10.1016/j.rcradv.2022.200086.
7. Barke, A.; Cistjakov, W.; Steckermeier, D.; Thies, C.; Popien, J.-L.; Michalowski, P.; Melo Pinheiro, S.; Cerdas, F.; Herrmann, C.; Krewer, U.; Kwade, A.; Spengler, T. S., Green batteries for clean skies: Sustainability assessment of lithium-sulfur all-solid-state batteries for electric aircraft. Journal of Industrial Ecology 2022, 1-16, DOI 10.1111/jiec.13345.
8. van der Hulst, M. K.; Huijbregts, M. A. J.; Loon, N. v.; Theelen, M.; Kootstra, L.; Bergesen, J. D.; Hauck, M., A systematic approach to assess the environmental impact of emerging technologies: A case study for the GHG footprint of CIGS solar photovoltaic laminate. Journal of Industrial Ecology 2020, 24, 1234-1249, DOI 10.1111/jiec.13027.
9. Piccinno, F.; Hischier, R.; Seeger, S.; Som, C., From laboratory to industrial scale: a scale-up framework for chemical processes in life cycle assessment studies. Journal of Cleaner Production 2016, 135, 1085-1097, DOI 10.1016/j.jclepro.2016.06.164.
10. Capello, C.; Hellweg, S.; Badertscher, B.; Hungerbühler, K., Life-Cycle Inventory of Waste Solvent Distillation: Statistical Analysis of Empirical Data. Environmental Science & Technology 2005, 39 (15), 5885-5892, DOI 10.1021/es048114o.
11. Zhang, X.; Wang, H.; Treyer, K., Development of unit process datasets. In *Life cycle inventory analysis*, Ciroth, A.; Arvidsson, R., Eds. Springer, Cham: 2021.
12. Desmarteau, D. D.; Witz, M., N-Fluoro-Bis(Trifluoromethanesulfonyl)imide. An improved synthesis. Journal of Fluorine Chemistry 1991, 52, 7-12, DOI 10.1016/S0022-1139(00)80317-6.
13. Siegemund, G.; Schwertfeger, W.; Feiring, A.; Smart, B.; Behr, F.; Vogel, H.; McKusick, B.; Kirsch, P., Fluorine Compounds, Organic. In *Ullmann's Encyclopedia of Industrial Chemistry*, 2016; p 473.
14. Wernet, G.; Hellweg, S.; Hungerbühler, K., A tiered approach to estimate inventory data and impacts of chemical products and mixtures. The International Journal of Life Cycle Assessment 2012, 17, 720-728, DOI 10.1007/s11367-012-0404-0.
15. Sutter, J. *Life cycle inventories of highly pure chemicals*; Ecoinvent: Uster, 2007.
16. Rösch, L.; John, P.; Reitmeier, R., Silicon Compounds, Organic. In *Ullmann's Encyclopedia of Industrial Chemistry*, 2000.

17. Wickerts, S.; Arvidsson, R.; Sandén, B. A.; Gregory, P.; Hou, L.; Albinsson, B., Prospective Life-Cycle Modeling of Quantum Dot Nanoparticles for Use in Photon Upconversion Devices. ACS Sustainable Chemistry & Engineering 2021, 9 (14), 5187-5195, DOI 10.1021/acssuschemeng.1c00376.
18. Friedrich, H.; Pfeffinger, J.; Leutner, B. Method for producing highly pure lithium salts. 2003.
19. Perez, W.; Suarez, C.; Bravo, M.; Barrientos, H. Lithium carbonate production from brine. 2011.
20. Daugs, E. D.; Appell, R. B.; Hippler, J. G.; Keen, B. T. Process for making polyethylene glycol compounds. EP 1797131 B1, 2012.
21. Clark Jr., E.; Straw, J. J. Process for producing sulfolane compounds. 1994.
22. Jun, S.; Joo, S. H.; Ryoo, R.; Kruk, M.; Jaroniec, M.; Liu, Z.; Ohsuna, T.; Terasaki, O., Synthesis of New, Nanoporous Carbon with Hexagonally Ordered Mesostructure. Journal of American Chemical Society 2000, 122 (43), 10712-10713, DOI 10.1021/ja002261e.
23. He, Z.; Alexandridis, P., Micellization Thermodynamics of Pluronic P123 (EO20PO70EO20) Amphiphilic Block Copolymer in Aqueous Ethylammonium Nitrate (EAN) Solutions. polymers 2017, 10 (32), 1-18, DOI 10.3390/polym10010032.
24. Moulijn, J. A.; Makkee, M.; Van Diepen, A. E., *Chemical Process Technology*. 2 ed.; John Wiley & Sons Ltd: West Sussex, 2013.
25. Noshay, A.; McGrath, J. E., A-B-A Triblock Copolymers. In *Block Copolymers. Overview and critical survey*, Academic Press: 1977.
26. Chordia, M.; Wickerts, S.; Nordelöf, A.; Arvidsson, R., Life cycle environmental impacts of current and future battery-grade lithium supply from brine and spodumene. Resources, Conservation & Recycling 2022, 187 (106634), DOI 10.1016/j.resconrec.2022.106634.
27. Chordia, M.; Nordelöf, A.; Ellingsen, L. A.-W., Environmental life cycle implications of upscaling lithium-ion battery production. The International Journal of Life Cycle Assessment 2021, 26, 2024-2039, DOI 10.1007/s11367-021-01976-0.
28. Jinasena, A.; Stokke Burheim, O.; Hammer Stromman, A., A Flexible Model for Benchmarking the Energy Usage of Automotive Lithium-Ion Battery Cell Manufacturing. batteries 2021, 7 (14), 1-21, DOI 10.3390/batteries7010014.
29. Heimes, H. H.; Kampker, A.; vom Hemdt, A.; Schön, C.; Michaelis, S.; Rahimzei, E. *Production of all-solid-state battery cells*; Frankfurt, 2018.
30. Schnell, J.; Günther, T.; Knoche, T.; Vieider, C.; Köhler, L.; Just, A.; Keller, M.; Passerini, S.; Reinhart, G., All-solid-state lithium-ion and lithium metal batteries – paving the way to large-scale production. Journal of Power Sources 2018, 382, 160-175, DOI 10.1016/j.jpowsour.2018.02.062.
31. Li, B.; Gao, X.; Li, J.; Yuan, C., Life Cycle Environmental Impact of High-Capacity Lithium Ion Battery with Silicon Nanowires Anode for Electric Vehicles. Environmental Science & Technology 2014, 48, 3047-3055, DOI 10.1021/es4037786.
32. Kolosnitsyn, V.; Karaseva, E. Battery electrode structure. US 9,219,271 B2, 2015.
33. Ellingsen, L. A.-W.; Majeau-Bettez, G.; Singh, B.; Srivastava, A. K.; Valøen, L. O.; Strømman, A. H., Life Cycle Assessment of a Lithium-Ion Battery Vehicle Pack. Journal of Industrial Ecology 2014, 18 (1), 113-124, DOI 10.1111/jiec.12072.
34. Ainsworth, D. In *Li-S Batteries for Energy Storage Applications*, Frontier Energy Storage Technologies and Global Energy Challenges, 2016; 2016.
35. Peters, J. F.; Weil, M., Aqueous hybrid ion batteries - An environmentally friendly alternative for stationary energy storage? Journal of Power Sources 2017, 364, 258-265, DOI 10.1016/j.jpowsour.2017.08.041.
36. Nordelöf, A.; Alatalo, M. *A Scalable Life Cycle Inventory of an Automotive Power Electronic Inverter Unit – Technical and Methodological Description, version 1.01*;

Department of Energy and Environment, Divisions of Environmental Systems Analysis & Electric Power Engineering, Chalmers University of Technology: Gothenburg, Sweden, 2018.
37. Nordelöf, A.; Alatalo, M.; Ljunggren Söderman, M., A scalable life cycle inventory of an automotive power electronic inverter unit—Part I: design and composition. The International Journal of Life Cycle Assessment 2019, 24 (1), 78-92, DOI 10.1007/s11367-018-1503-3.
38. Nordelöf, A., A scalable life cycle inventory of an automotive power electronic inverter unit—Part II: manufacturing processes. The International Journal of Life Cycle Assessment 2019, 24 (4), 694-711, DOI 10.1007/s11367-018-1491-3.
39. Pellow, M. A.; Ambrose, H.; Mulvaney, D.; Betita, R.; Shaw, S., Research gaps in environmental life cycle assessments of lithium ion batteries for grid-scale stationary energy storage systems: End-of-life options and other issues. Sustainable Materials and Technologies 2020, 23 (e00120), 1-17, DOI 10.1016/j.susmat.2019.e00120.
40. Zhang, Z.; Ding, T.; Zhou, Q.; Sun, Y.; Qu, M.; Zeng, Z.; Ju, Y.; Li, L.; Wang, K.; Chi, F., A review of technologies and applications on versatile energy storage systems. Renewable and Sustainable Energy Reviews 2021, 148 (111263), 1-31, DOI 10.1016/j.rser.2021.111263.
41. Battke, B.; Schmidt, T. S.; Grosspietsch, D.; Hoffmann, V. H., A review and probabilistic model of lifecycle costs of stationary batteries in multiple applications. Renewable and Sustainable Energy Reviews 2013, 25, 240-250, DOI 10.1016/j.rser.2013.04.023.
42. Berg, H., *Batteries for Electric Vehicles. Materials and Electrochemistry*. Cambridge University Press: Cambridge, United kingdom, 2015.
43. da Silva Lima, L.; Quartier, M.; Buchmayr, A.; Sanjuan-Delmás, D.; Laget, H.; Corbisier, D.; Mertens, J.; Dewulf, J., Life cycle assessment of lithium-ion batteries and vanadium redox flow batteries-based renewable energy storage systems. Sustainable energy technologies and assessments 2021, 46 (101286), 1-13, DOI 10.1016/j.seta.2021.101286.
44. Tillman, A.-M.; Nordelöf, A.; Grunditz, E.; Lundmark, S.; Alatalo, M.; Thiringer, T.; Ljunggren, M. *Elmaskiner för fordon i en cirkulär ekonomi. Design för miljö- och resurseffektivitet och krav på End-of-Life system*; Chalmers university of technology: Gothenburg, 2020.
45. Schwich, L.; Sabarny, P.; Friedrich, B., Recycling Potential of Lithium–Sulfur Batteries—A First Concept Using Thermal and Hydrometallurgical Methods. Metals 2020, 10 (1513), 1-19, DOI 10.3390/met10111513.
46. Huang, X.; Xue, J.; Xiao, M.; Wang, S.; Li, Y.; Zhang, S.; Meng, Y., Comprehensive evaluation of safety performance and failure mechanism analysis for lithium sulfur pouch cells. Energy Storage Materials 2020, 30, 87-97, DOI 10.1016/j.ensm.2020.04.035.
